# Neuron anatomy structure reconstruction based on a sliding filter

**DOI:** 10.1186/s12859-015-0780-0

**Published:** 2015-10-24

**Authors:** Gongning Luo, Dong Sui, Kuanquan Wang, Jinseok Chae

**Affiliations:** 1 Research Center of Perception and Computing, School of Computer Science and Technology, Harbin Institute of Technology, Harbin, China; 2 Department of Computer Science and Engineering, Incheon National University, Incheon, Korea

**Keywords:** Neuron anatomy structure reconstruction, Radius estimation, Sliding filter, Open curve snake model

## Abstract

**Background:**

Reconstruction of neuron anatomy structure is a challenging and important task in neuroscience. However, few algorithms can automatically reconstruct the full structure well without manual assistance, making it essential to develop new methods for this task.

**Methods:**

This paper introduces a new pipeline for reconstructing neuron anatomy structure from 3-D microscopy image stacks. This pipeline is initialized with a set of seeds that were detected by our proposed Sliding Volume Filter (SVF), given a non-circular cross-section of a neuron cell. Then, an improved open curve snake model combined with a SVF external force is applied to trace the full skeleton of the neuron cell. A radius estimation method based on a 2D sliding band filter is developed to fit the real edge of the cross-section of the neuron cell. Finally, a surface reconstruction method based on non-parallel curve networks is used to generate the neuron cell surface to finish this pipeline.

**Results:**

The proposed pipeline has been evaluated using publicly available datasets. The results show that the proposed method achieves promising results in some datasets from the DIgital reconstruction of Axonal and DEndritic Morphology (DIADEM) challenge and new BigNeuron project.

**Conclusion:**

The new pipeline works well in neuron tracing and reconstruction. It can achieve higher efficiency, stability and robustness in neuron skeleton tracing. Furthermore, the proposed radius estimation method and applied surface reconstruction method can obtain more accurate neuron anatomy structures.

**Electronic supplementary material:**

The online version of this article (doi:10.1186/s12859-015-0780-0) contains supplementary material, which is available to authorized users.

## Background

Neuron morphology and structure information is critical for neuroscience research. Hence, reconstructing the entire anatomy structure of a neuron is an essential task in the field of neuron informatics [1, 2]. However, reconstructing the anatomy structure of a neuron artificially is labor intensive. Efficient, advanced methods for anatomy structure reconstruction of neurons are greatly demanded in this field. Specifically, with the rapid development of microscopic imaging technology, a wide range of scales of bio-images can be obtained, which is helpful for us to develop new methods and algorithms to meet the needs in neuroscience research [3, 4]. The reconstructed digital neuron structure, including axons and dendrites as well as thickness information, can be used in conjunction with electrophysiological simulations to determine the complex mechanisms of the nervous system [5, 6].

The computer-aided manual neuron reconstruction method was first proposed in 1965 and was achieved by a biologist using a microscope [7]. Following this milestone, numerous algorithms and open softwares were introduced to reduce manual labor consisting of the boring task of tracing and analysis [8–11], but most of them were still limited to semi-automation and required manual validation by experts to achieve accurate reconstruction of whole neurons. Hence, the lack of powerful and effective computational tools for automatically reconstructing neuron cells has emerged as a major technical bottleneck in neuroscience research. This problem motivated the DIgital reconstruction of Axonal and DEndritic Morphology (DIADEM) challenge [12] and BigNeuron project [13, 14], which began in 2010 and 2015 respectively. They provided an open-source platform for researchers from all over the world and aimed to promote the development of computer algorithms for reconstructing the full anatomy structure of neurons. The data sets from DIADEM are most widely used in the domain of neuron reconstruction to date. However, the BigNeuron proposed some new challenges for the further research in the field of neuron reconstruction.

Generally speaking, before the DIADEM project, the neuron tracing methods were categorized into several types: shortest path methods [15, 16], minimum spanning tree methods [17, 18], sequential tracing methods [19, 20], skeletonization methods [21, 22], neuromuscular projection fiber tracing methods [23–25] and active contour-based tracing methods [26–28]. Based on these methods, some new improved methods were proposed [29]. The DIADEM final listed five well-performed algorithms: the model-based method [30], geometry-based method [31], probabilistic approach-based method [32], open snake-based method [33] and cost minimization trees-based approach [34]. In the model-based method, Myers’s team employed the idea of shortest paths to refine local tracing, which is based on the model of Al-Kofahi [19] and a formal tube model. This pipeline can reconstruct the neuron from raw or preprocessed images [30]. In the geometry-based method, Erdogmus’s team introduced a principal curve to represent the skeleton of axons, and they then extracted the topology information using a recursive principal curve tracing method [31]. In the probabilistic approach-based method, Gonzalez’s team built a set of candidate trees to choose the best one by a global objective function, which combined geometric priors from image evidence [32]. In the open snake-based method, Roysam’s team proposed a three dimensional open curve snake model that was initiated automatically by a set of skeletons from binary images generated by the 2-D graph cut pre-segmentation method, and the snake curve could be stretched bi-directionally along the centerline to trace the neuron cell structure [33]. Stepanyants’s team proposed trees-based method, which can merge individual branches into trees based on a cost minimization strategy [34]. After the DIADEM final, Liu’s group proposed a 3D neuronal morphology reconstruction method based on the augmented ray burst sampling method [35]. This method consisted of a single step to achieve the tracing and reconstruction, in which the centerline extraction or the extra radius estimation was unnecessary but the first seed must be set artificially. Peng’s team proposed series of efficient methods for neuron reconstruction, such as an anisotropic path searching method [36], an all-path pruning method [37], a hierarchical-path pruning method based on a gray-weighted image distance-tree [38], an automatic distance-field neuron tracing method based on global threshold foreground extraction [39], a smart tracing method based on machine learning [40] and a method based on reverse mapping and assembling of 2D projections [41]. These methods can work well with the neuron center lines tracing under the complex and noisy background. Kakadiaris’s team proposed a learning 3D tubular models-based method, which can use a morphology-guided deformable model to extract the dendritic centerline and use minimum shape-cost tree to represent the neuron morphology [42]. In addition, to achieve more accurate neuron tracing results, some open source softwares have been developed, such as flNeuronTool [35], FarSight [33], V3D [10], and Vaa3D [43, 44]. Along with all the existing algorithms, these open source softwares also promote the development of neuron reconstruction.

Despite the large number of proposed neuron tracing algorithms mentioned above, few methods can automatically reconstruct the complete and detailed neuron morphology, including complex dendritic and axonal arbors and variable thickness information. Moreover, because of the limited computer power, the automatic and accurate reconstruction of neuron anatomy structure is still a significant challenge.

In this paper, we propose a new 3D seed detection method based on Sliding Volume Filter (SVF) to initialize our framework, and we designed an open curve snake model combined with a SVF external force for centerline extraction and tracing. This open curve snake model has higher efficiency in the convergence of endpoints and detection of branch collision. In addition, radius estimation is another critical problem in neuron reconstruction, and accurate radius estimation can benefit simulation and functional research. Hence, this paper also proposes a new radius estimation method based on a 2D sliding band to estimate the radius of a neuron. The proposed radius estimation method can fit the real edges of neuron non-circular cross-sections better than previous methods. Finally, a surface reconstruction method based on contour lines is adopted to reconstruct detailed neuron morphology.

## Methods

**Fig. 1:**
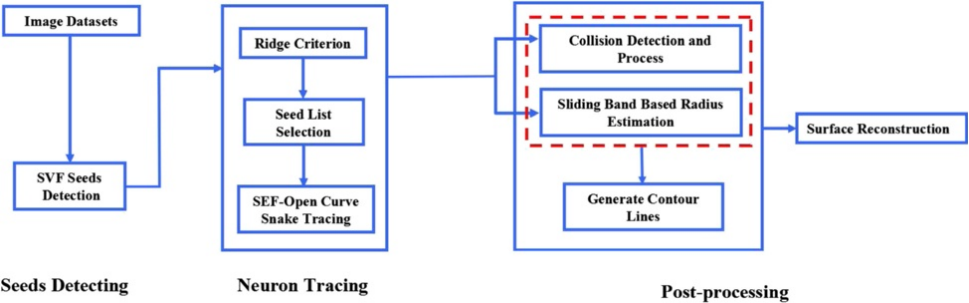
Pipeline of neuron anatomy structure reconstruction

As shown in Fig. 1, some critical steps, such as seeding, tracing, radius estimating and surface reconstruction, are included in the pipeline of our protocol. The details of every critical step will be explained.

### Seed detection

Seed detection is a critical procedure in the open snake-based tracing protocol, and an ideal seed list can ensure tracing accuracy. The proposed seeding method includes the following two stages:

1. We used the proposed SVF based method to select coarse seeding points in the interior of neuron cells.
2. The ridge criterion was used to achieve the further filter to obtain better seeding points, which are always near the center of the neuron cell.

#### A. SVF-Based seeding

In the field of computer vision and image processing, the convex region is defined as follows:

- A rounded convex region is a region with higher intensity in the center than the edge, and the gradient vectors of this region point to its center.
- A tube-like convex region is a region with higher intensity along its centerline than the edge, and the gradient vectors point to the centerline from the edge.

Quelhas’s group proposed a 2D Sliding Band Filter (SBF) for cell nucleus detection based on the characteristic of a rounded convex region [45]. In the data sets of microscopic imaging, a 3D neuron cell has not only a tube-like convex region but also a non-circular cross-section. Given these two characteristics, we extended the SBF into 3D space and designed a Sliding Volume Filter (SVF) to enhance the tube-like convex region for seed detection of neuron volume data.

**Fig. 2:**
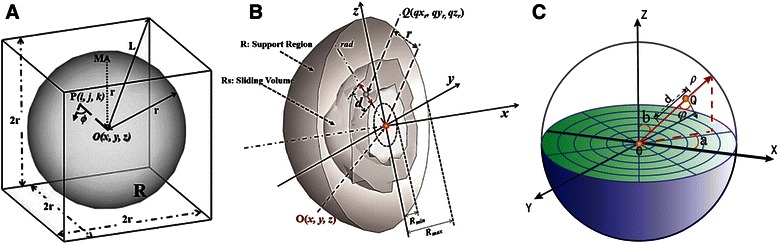
Scheme of Spatial Convergence Index. **a** The model of 3D spatial convergence index. **b** The model of 3D sliding volume filter in y-z plate section. **c** the discretization calculation of SVF using the polar coordinates

To explain the calculation of SVF, we first explained the Voxel Convergence Index (VCI). As shown in Fig. 2a, *O* is an interested voxel in 3D volume datasets with its coordinate located at (*x, y, z*). A sphere support region *R* is located around the center *O*, and *P* are the voxels in the support region *R* except at *O*, whose coordinate is (*i, j, k*). ϕ(*i, j, k*) is the angle between *PO* and the gradient vector direction. The VCI of *P* is defined as follows:

1 $$ VC{I}_p\kern0.5em =\kern0.5em  \cos {\varphi}_{\left(i,j,k\right)} $$

Figure 2b and c show the calculation scheme of the sliding volume filter in a support region *R* whose radius is *rad*. To finish the discretization computation efficiently, the polar coordinate is introduced into this scheme, and the SVF is calculated as

2 $$ SV{F}_O\kern0.5em =\kern0.5em \frac{1}{M}{\displaystyle \sum_{a=0}^{2\pi }{\displaystyle \sum_{b=0}^{\pi}\underset{R_{\min }<r<{R}_{\max }}{ \max}\left({\scriptscriptstyle \frac{1}{d+1}}{\displaystyle {\sum}_{r-d/2}^{r+d/2}VC{I}_Q}\right)}} $$

with

3 $$ VC{I}_Q\kern0.5em =\kern0.5em  \cos \varphi \left(q{x}_{\rho },q{y}_{\rho },q{z}_{\rho}\right)\kern0.5em =\kern0.5em  \cos \varphi \left(\rho \sin b \cos a,\rho \sin b \sin a,\rho \cos b\right) $$

where *M* is the number of support region lines radiating from the center pixel *O(x, y, z)*, *ρ* denotes the radial coordinate, *a* and *b* stand for the angular coordinates, *d* is the thickness of sliding volume, *r* is the center position of the sliding volume in the support region line ranging from *R*_*min*_ to *R*_*max*_, *Q* is the points between [*r−d/2,r + d/2*], and *φ*(*qx*_*ρ*_, *qy*_*ρ*_, *qz*_*ρ*_) is the angle between the gradient vector at *Q* and the direction of *QO*. Additionally, the angles *a* ∈ [0, 2*π*] and *b* ∈ [0, *π*] are divided into 2 *L* parts and *L* parts, respectively. Thus, *M = 2 L* ^*2*^. Specially, the number of parts of *L* determines the accuracy and efficiency of computation.

**Fig. 3:**
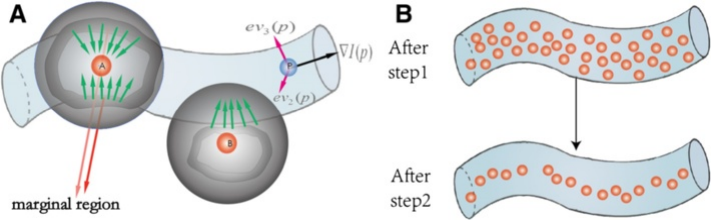
Scheme of computation of Sliding Volume Filter, as well as the selection of seed points. **a** The procedure of seed points filtrate, after the SVF and ridge criterion, proper seed points are chosen. **b** The seeding points selection after step1 and step2

After the SVF was applied to the neuron volume data for seed detection voxel by voxel, we selected the voxels as the raw seeds whose SVF response values are higher than the threshold *T*. Notably, there are more gradient vectors that point to the center of a tube-like structure in the marginal regions than in the other regions [45]. Hence, the sliding volumes of support regions of interior points are more likely to converge in the marginal regions. As shown in Fig. 3a, the voxel *A* in the interior of the nerve is more likely to be selected as a raw seed than the external voxel *B*. Because *A* has a higher SVF response value than voxel B, the orientations of gradient vectors in the sliding volumes of support region of A are more likely to point to A. However, the orientations of gradient vectors in the sliding volumes of the support region of B are not consistent and sometimes point away from B. Moreover, a nerve cell is not a uniform tube-like structure but instead has variable thickness. Therefore, SVF is the proper filter for raw seed selection.

#### B. Ridge criterion

The Aylward’s ridge criterion method was applied to the raw seeds for the final seed choice [20]. As shown in Fig. 3a, *I* is the volume data set, ∇*I*(*p*) is the gradient vector at voxel *p* with its coordinate *(x, y, z)* in *I*, and *ev*_1_, *ev*_2_ and *ev*_3_ are the eigenvectors computed from Hessian matrix of *I. ev*_1_(*p*) is the principle direction along the center lines of the tube-like structure, and *ev*_2_(*p*) and *ev*_3_(*p*) are the other two orthogonal eigenvectors. The seeds near the center of the tube-like structcure meet the condition of Eq. 4.

4 $$ e{v}_2(p)\cdotp \nabla I\kern0.5em (p)<0.001\kern0.5em \mathrm{and}\kern0.5em e{v}_3(p)\cdotp \nabla I(p)<0.001 $$

The raw seeding points were further chosen according to the ridge criterion Eq. 4. As shown in Fig. 3b **,** after the steps of SVF and ridge criterion, the proper seeds near the center line of the nerve are chosen and included in the seed list, in which the seed points are sorted by the response values. Simultaneously, response values from SVF are used to enhance the intensity of voxels in the original data, which benefits the deformation of the open curve model in the following step. The SVF volume enhancement method is denoted as

5 $$ {I}_{SVF}(p)\kern0.5em =\kern0.5em 15\sqrt{ \min \left[I(p)*\left(1+SVF(p)\right),255\right]} $$

where *I*_*SVF*_(*p*) is the intensity of point *p* after SVF enhancement, *I*(*p*) is the intensity of point *p* before SVF enhancement, and *SVF*(*p*) is the SVF value of point *p*.

#### SEF-OCS Neuron tracing

Tracing the full neuron skeleton is still a challenging task in neuron science, although many methods have been proposed. In this section, a new tracing model is proposed called an SVF external force open curve snake (SEF-OCS, SEF-Open Curve Snake). The open curve snake model was initially applied to automated actin filament segmentation and tracking [33, 46]. Extended the application of the open curve snake model to neuron tracing. However, the computation was tedious in the tracing framework of [33], especially in the step of branch detection. The proposed SEF-OCS includes three parts: open curve deformation, curve extension, and collision detection.

#### A. Open curve deformation

This model is a parametric open curve model, and the total snake energy can be defined as

6 $$ {E}_{Total}\kern0.5em =\kern0.5em {E}_{Internal}+{E}_{External} $$

*E*_*Total*_ is the total image energy combined with internal energy and external energy. This model is a traditional deformable model, which resembles previous work in [16]. The open snake model is a parametric curve, *c*(*s*) = (*x*(*s*), *y*(*s*), *z*(*s*)), *s*∈[0, 1], and the snake internal and external energy are defined as follows:

7 $$ {E}_{Internal}={\displaystyle {\int}_0^1\alpha }{\left|{c}_s(s)\right|}^2+\beta {\left|{c}_{ss}(s)\right|}^2ds $$

8 $$ {E}_{External}\kern0.5em =\kern0.5em {\displaystyle {\int}_0^1{E}_{im}\left(c(s)\right)+{E}_{str}\left(c(s)\right)\kern0.5em }ds $$

with

$$ \begin{array}{l}\nabla {E}_{im}=-\nabla {I}_{SVF}(p)\\ {}\nabla {E}_{str}\left(c(s)\right)=-\left\{\begin{array}{c}\hfill -{\scriptscriptstyle \frac{c_s(s)}{\left\Vert {c}_s(s)\right\Vert }}\left\Vert \frac{\sqrt{\left|{\lambda}_2{\lambda}_3\right|}}{\left|{\lambda}_1+0.01\right|}-1\right\Vert \kern1.25em s=0\hfill \\ {}\hfill {\scriptscriptstyle \frac{c_s(s)}{\left\Vert {c}_s(s)\right\Vert }}\ \left\Vert \frac{\sqrt{\left|{\lambda}_2{\lambda}_3\right|}}{\left|{\lambda}_1+0.01\right|}-1\right\Vert \kern1.49em s=1\hfill \\ {}\hfill 0\kern7.75em s\in \left(0,1\right)\hfill \end{array}\right.\end{array} $$

**Fig. 4:**
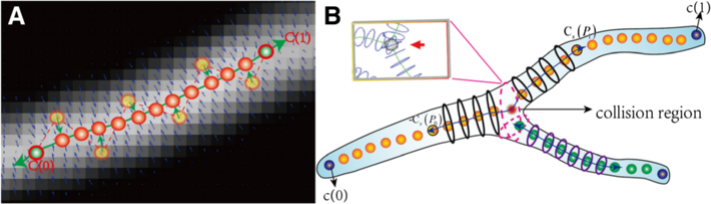
Scheme of SEF open curve snake model. **a** The open curve is driven to the center of neuron by external force in the volume after SVF. **b** The procedure of open snake curve extension and collision detection in the branching region

In Eq. 7, *α* and *β* are the “elasticity coefficient” and “stiffness coefficient”, respectively, in internal energy, and they can control the regularity of the curve in the process of evolution. In Eq. 8, the external energy term is used to make the snake deform near the center line of the neuron and stretch the endpoints to the tail of the neuron. ∇*E*_*im*_ is the negative normalized Gradient Vector Flow (GVF) of the volume data enhanced by SVF, *p* signifies point (*x(s), y(s), z(s)*) on the open curve, and *I*_*SVF*_ is the volume after SVF enhancement in this paper. Instead of the original 3D image GVF, we calculated the GVF of *I*_*SVF*_. The SVF can enhance the tube-like convex region to smooth the GVF. As shown in Fig. 4a, the blue arrows show examples of gradient vectors from the volume enhanced by *SVF*. The vectors point toward the centers of neurons, which can make the seed points (the yellow points in Fig. 4a) move to the center position (the position of the red points in Fig. 4a). Specifically, the stretching force ∇*E*_*str*_(*c*(*s*)) is only implemented to the final endpoints *c* (0) and *c* (1). The *c*_s_(*s*)/||*c*_s_(*s*)|| denotes the direction of the stretching force. The value $$ \left\Vert \sqrt{\lambda_2{\lambda}_3}{\scriptscriptstyle \raisebox{1ex}{$$}\!\left/ \!\raisebox{-1ex}{$$}\right.}\left({\lambda}_1+0.01\right)-1\right\Vert $$ is used to measure the tube-like level around the end point. When a curve reaches the end of a neuron, the end points will lose the tube-like characteristic. Hence, ∇*E*_*str*_(*c*(*s*)) approaches zero, and the open active curve converges. According to a large number of experiments, this strategy is not only efficient and reliable but also can avoid the leakage of the neuron boundary. To minimize the energy function *E*_*Total*_, the points on the snake curves are updated as:

9 $$ \begin{array}{l}{x}_t={\left(\gamma {I}_{SVF}+A\right)}^{-1}\left(\gamma {x}_{t-1}+\partial {E}_{ext}\left({x}_{t-1},{y}_{t-1},{z}_{t-1}\right)/\partial x\right);\\ {}{y}_t={\left(\gamma {I}_{SVF}+A\right)}^{-1}\left(\gamma {y}_{t-1}+\partial {E}_{ext}\left({x}_{t-1},{y}_{t-1},{z}_{t-1}\right)/\partial y\right);\\ {}{z}_t={\left(\gamma {I}_{SVF}+A\right)}^{-1}\left(\gamma {z}_{t-1}+\partial {E}_{ext}\left({x}_{t-1},{y}_{t-1},{z}_{t-1}\right)/\partial z\right);\end{array} $$

where the parameters *t* and *γ* control the iteration numbers and size of the step at each iteration, respectively. The iterations are stopped when *t* reaches the threshold of the max iteration number.

#### B. Curve extension

The initial open snake curve is formed by three points (fewer than three points will not be traced as a branch of neuron). The first point *p* has the best response value in the seed list, and the other two points are generated by extending along the first principal direction to *ev*_1_(*p*) and −*ev*_1_(*p*). As shown in Fig. 4b, along with the open snake curve moving to the center of neuron, it also extends toward the two inverse tangential directions, *c*_s_(*p*_0_) and −*c*_s_(*p*_1_), in which the *p*_0_ and *p*_1_ are the two temporary endpoints. During the procedure of extension, the seed points belonging to one curve were labelled with new values (the default value is zero) in accord with the ID of the curve. For example, in Fig. 4b the yellow points and green points belong to different curves.

#### C. Collision detection

Neurons have many branches, especially in the dendrite region. Hence, detecting branching points and handling collision are essential. In the proposed scheme, two types of collision exist in the collision region and are shown in Fig. 4b. The first collision is branching point collision, which occurs when the open snakes reach a seed point whose value is not zero, and this point is recorded as the branching point (pink point in Fig. 4b). This branching point detection strategy is based on labelling seeds and is highly efficient. It also can handle the second type of collision, contour lines collision. The contour lines coming from the following step of radius estimation are the foundation of neuroanatomy reconstruction. However, due to the ambiguity of radius estimation in the collision region, the contour lines from two curves easily intersect. In Fig. 4b, this situation is illustrated in the imaginary pink circle and the embedded image, which is an experimental result in the branching region. This collision will influence the accuracy of the following reconstruction algorithm. Hence, a backoff strategy is proposed to avoid contour line collision. First, radius estimation in the branching points will not be executed. Second, if an extending curve reaches the branching point, it will be cut back the length of *D*, which is usually set as double the average estimated radius of the current curve.

In other words, the imaginary pink circle is not necessary in radius estimation because the following reconstruction algorithm would interpolate the information using triangular meshes automatically. Finally, the tracing algorithm ends when all the seed points are traversed.

The entire tracing algorithm procedure is shown as follows:

In summary, compared to the open snake method in [33], we improved this model in the following three aspects. First, the volume after SVF enhancement has more straightforward gradient vectors, which point to the center line of the neuron and can be used in driving the initial lines to the center of the neuron. Second, the proposed method can cut down the computation of the stretching force of end nodes. Third, in the step of collision detection, compared to the method based on labelling neighbor voxels, the method based on labelling seeds has higher detection efficiency and benefits the following reconstruction procedure.

#### Radius estimation

**Fig. 5:**
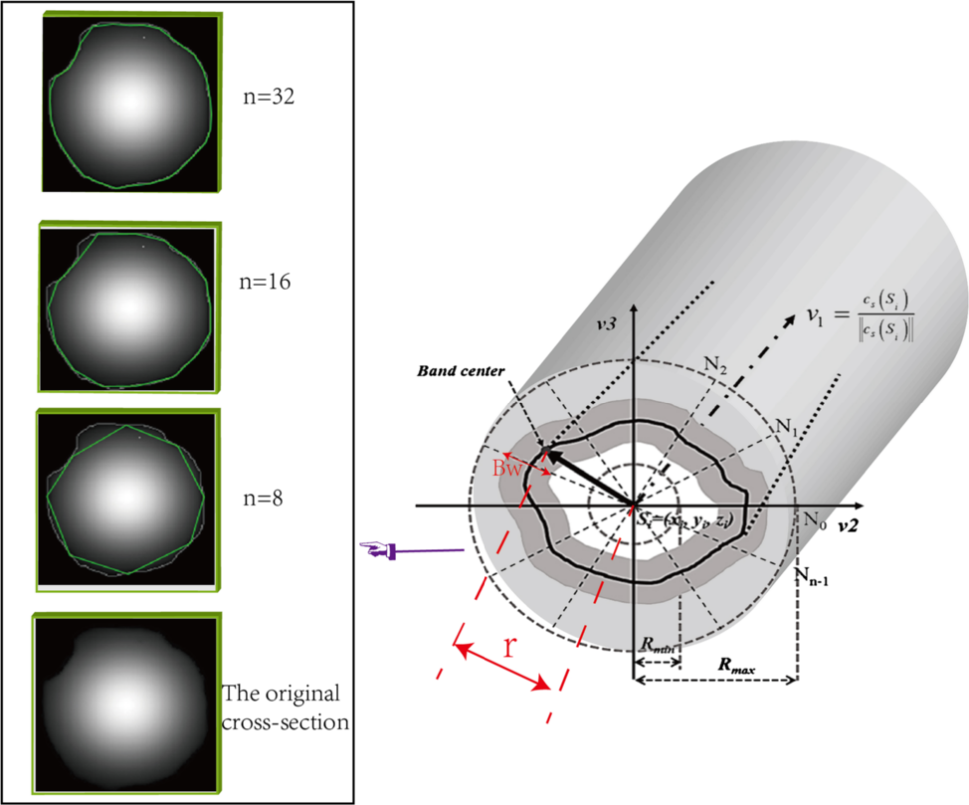
Illustration of radius estimation of the neuron cross-section. The left embedded image shows the real cross-section of neuron, and the estimation result with different parameters. And in the right image *v*_*1*_ is the tangential direction in *S*_*i*_, *v*_*2*_ and *v*_*3*_ are the orthogonal vectors which define the cross-section

Radius estimation is another critical task in neuron anatomy reconstruction, and it can provide more quantitative information for neuroscience research. Peng, Aylward, and Wang had proposed some radius estimation methods [16, 20, 33], but most of them are based on the assumption that the neuron have a uniform tube-like structure, whose cross-sections are regular circles. However, the real cross-sections are not regular circles, as shown in the embedded image of Fig. 5. To reconstruct the neuron morphological structure more accurately, fitting the real edge of the neuron cell is achieved by a new proposed radius estimation method based on a 2D Sliding Band Filter (SBF) [45]. The SBF can converge on the real edge of a neuron cross-section that has the rounded convex region in [45].

Figure 5 shows the scheme of the radius estimation method based on a 2D sliding band filter. We could obtain the cross-section according to the normal vector *v*_*1*_, which points to the tangential direction of the open curve. Additionally, the *v*_*2*_ and *v*_*3*_ are the orthogonals in the cross-section. To obtain an accurate estimation of neuron cross-section, *n* radiuses radiating from the center point *S* on the snake curve are estimated as different lengths. The radius lengths are equal to *r* in the Eq. 10 with the maximum SBF response value.

10 $$ B\left(n,r\right)=\underset{R_{\min }<r<{R}_{\max }}{ \max }SB{F_r}^n\left({x}_r^n,{y}_r^n,{z}_r^n\right) $$

with,

$$ SB{F}_r^n\left({x}_r^n,{y}_r^n,{z}_r^n\right)\kern0.5em =\kern0.5em {\scriptscriptstyle \frac{1}{d+1}}{{\displaystyle {\sum}_{r-d/2}^{r+d/2}VCI}}_P $$

where *B* is the boundary points on the cross-section, which are at the centers of sliding bands and will be used to fit the real edge. (*x*_*r*_^*n*^, *y*_*r*_^*n*^, *z*_*r*_^*n*^) are the spatial coordinates of *S*. The computation method of *SCI* in point *P*, which is in the range of [*r−d/2, r + d/2*], has been introduced in Eq. 4. *d* is the width of the sliding band, *r* is the distance between *B* and the center point *S*, and it can slide in the range of [*R*_*min*_ *, R*_*max*_] to obtain the optimal position of *B* with the maximum SBF response value. The boundary points *B* can be connected clockwise to fit the edge of the neuron cell.

In the proposed method, the parameter *n* is related to the accuracy of radius estimation. As shown in the embedded image of Fig. 5, the larger *n* is, the more accurate the cross-section fitting will be. However, considering the efficiency and accuracy in the actual application, the parameter *n* should be adjusted flexibly.

#### Neuron surface reconstruction

Most of the traditional neuron reconstruction methods were based on the fast marching method and some supplemental processes for connecting different fragments [33, 47]. However, in this paper, Liu’s non-parallel contour lines surface reconstruction method is employed for surface reconstruction [48], considering the non-parallel characteristic of circles generated from previous steps. On the premise of an accurate description of the entire neuron anatomy structure, this method is efficient. Although this method had been widely used in other biological models, it has rarely been used in neuron model reconstruction. The generated mesh model of the neuron can benefit the future finite element mesh subdivision and simulation.

**Fig. 6:**
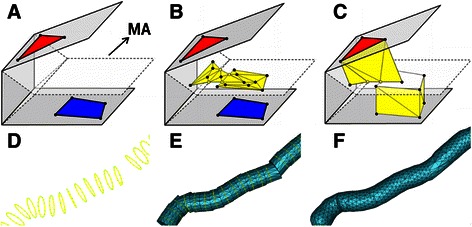
Process of contour reconstruction. **a** Construction of projective plate MA [48]. **b** Projection of points and lines on MA [48]. **c** Triangulation of adjacent contour lines [48]. **d** The contour lines of neuron cell. **e** The initial surface from triangulation of adjacent contour lines. **f** The final surface model after smoothing

**Fig. 7:**
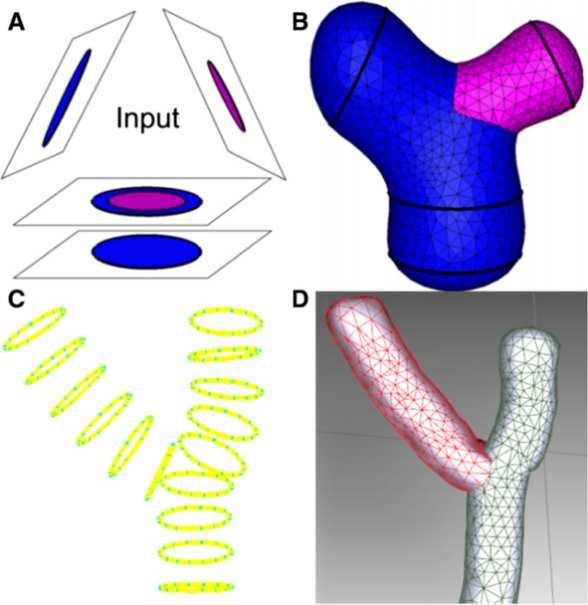
Process of neuron reconstruction in branching region. **a** The input branching data from [48]. **b** The reconstruction result of input data of (**a**). **c** The input data of neuron contour lines. **d** The reconstruction result of (**c**), in which different branches are labelled by different colors

Figure 6 shows the scheme of Liu’s method. First, it constructs *medial axes* (MA) between adjacent contour lines (Fig. 6a). Second, the points and lines from different contours are projected on the MA (Fig. 6b). Third, triangular meshes are used to connect the curve networks to their projection points on the MA (Fig. 6c). Finally, the surface meshes, which are connected with different contour lines, are formed as the boundaries between neighboring compartments [48]. To obtain a smoother neuron surface, we use a surface diffusion smoothing algorithm to minimize the curvature of the model surface to obtain a smooth 3D model [49]. As shown in Fig. 6, the initial neuron surface (Fig. 6e) is formed by contour lines (Fig. 6d) which are obtained by radius estimation, and the final smooth neuron surface is shown in Fig. 6f. In addition, the most outstanding advantage of Liu’s method is that it can automatically handle branch reconstruction, especially of circles without intersections in the branching region (the intersection problem was resolved through removing the collisions of circles in the SEF-OCS neuron tracing step). As shown in Fig. 7, in the branching region, two branches could be automatically reconstructed with different label colors.

## Results and discussion

### Parameters

We validated and evaluated the steps of seeding, tracing, radius estimation and neuron reconstruction in the proposed method using synthetic data and real data from the DIADEM challenge [12] and parts of datasets from BigNeuron project [13, 14]. All of the experiments were performed on an ordinary computer (Intel Core i5 3.2 CPU, NVIDIA GeForce GTX 960, 8 GB RAM, Windows 7). The proposed algorithm was developed using C++ language. In addition, to compare the other methods equally, we did not adopt any manual interactive operations shown in the Fig. 1, such as preprocessing, picking and expending seeds, checking and validating data, tracing editing, branch refining, and rooting setting, although these operation can improve the result of neuron tracing.

According to the image data sets (DIADEM challenge and BigNeuron project) chosen in this paper, Table 1 shows the parameters selected for the following experiments. Some experimental parameters such as *d*, *T*, *L*, *γ*, *ɑ*, and *β* remain constant for the following experiments. Other parameters such as *rad*, *R*_*max*_, *R*_*min*_ *, n* and *t* could be adjusted for optimal results.

**Table 1:** Parameter selection

Parameter		Value/range	Notes
Sliding filter	*rad*	20–30 (voxel)	This parameter determines the size of the support region of SVF and SBF*.* Too large or too small of a region will lead to computation-intensive processes or a reduction in the quality of seeding point selection and radius estimation.
	*d*	8 (voxel)	This parameter is the width of the sliding volume and sliding band, and it remains constant in the following experiments.
	*L*	20	This parameter remains constant in the following experiments. Too large of an *L* value will lead to computationally intensive processes.
	*R*_*max*_	*rad-d/2*	This parameter is same in seeding and radius estimation.
	*R*_*min*_	(*d/2, rad-d/2*)	This parameter is same in seeding and radius estimation. If the datasets are generated from bright field microscopy, a larger *R*_*min*_ should be set. Otherwise, a smaller *R*_*min*_ should be set.
	*n*	8–32	The larger the *n* value, the better the radius estimation and the lower the computation efficiency.
	*T*	0.7	This parameter is the threshold used to select the coarse seeding points, and it remains constant in the following experiments.
SEF-Open curve snake	*t*	10–25	In most experiments, 10 is sufficient for the curve evolution in the GVF field of volume after SVF enhancement.
	γ	2	This parameter controls the steps of evolution, and it is a fixed constant in following experiments.
	ɑ	0.8	This parameter representing the elasticity coefficient remains constant in the following experiments. A larger ɑ value makes the open snake smoother.
	β	β(s) = 0.2, 0 < s < 1	This parameter of the stiffness coefficient remains constant in the following experiments. A larger β value makes the snake stiffer.
		β(s) = 0, s = 0 or s = 1	

### Seeding

Generally speaking, an ideal seed point is located in the neuron body of the foreground, and its position is near the center of the neuron cell’s cross-section. To quantify the performance of SVF seed detection, we evaluated the seeding method using an artificial helix dataset and the real dataset of the DIADEM challenge [12], according to following point deviation measurement principle:

11 $$ \mathrm{D}\left({\mathrm{P}}_S,{\mathrm{P}}_g\right) = \kern0.5em \frac{1}{N}\ {\displaystyle \sum_{p\in {\mathrm{P}}_S}{d}_{\min}\left(p,{P}_g\right)} $$

where P_g_ denotes point sets in the gold standard, P_s_ denotes point sets generated by tested methods and *N* is the number of points in P_s_. *d*_*min*_ is the distance between a seeding point and its nearest point in the gold standard.

We compared the proposed seeding methods with the two most widely used seeding point detection methods, the global threshold method [18] and the LoG threshold method [50]. We set the parameters *rad* = 20, *R*_*max=*_16, and *R*_*min=*_5 in this experiments.

In the proposed *SVF* filter method, compared with two other methods, most of the seed points are in the interior of the neuron body. Table 2 shows the seed deviation results of the artificial helix body and OP_1, in which the *SVF* seeding method can achieve the lowest deviation.

**Table 2:** Comparisons of different seeding methods on test datasets

Dataset	Method	Detected seeds	Seeds in foreground	Seeds in background	Deviation (voxels)
Artificial helix body	Global Threshold Seeding	47	44	3	3.7
	LoG Threshold Seeding	33	30	3	3.5
	SVF Seeding	67	67	0	**1.7**
OP_1	Global Threshold Seeding	909	763	146	3.2
	LoG Threshold Seeding	829	704	125	2.9
	SVF Seeding	727	722	5	**1.5**

The best values are highlighted with bold letters

**Fig. 8:**
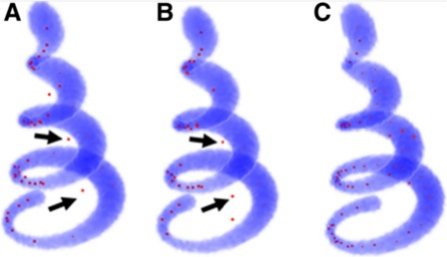
Comparison of seeding method on test dataset; **a** The seeds detection result of global threshold method. **b** The seeds detection result of LoG threshold method. **c** The seeds detection result of SVF method

**Fig. 9:**
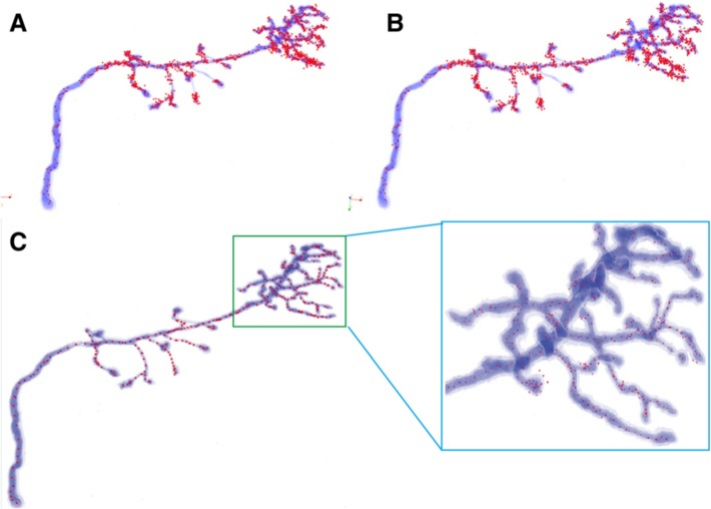
Comparison of seeding method on drosophila olfactory axonal data sets (OP_1). **a** The seeds detection result of global threshold method. **b** The seeds detection result of LoG threshold method. **c** The seeds detection result of SVF method and the enlargement image in intensive region of dendrites

As shown in Fig. 8, some seeds fall outside of the artificial helix body, which are detected by traditional threshold methods and highlighted with arrows. Figure 9 shows the seeding results in drosophila olfactory axonal datasets (OP_1 of DIADEM challenge), which are generated by the three mentioned methods. These results also suggest that our method is better than the two other seed detection methods.

**Fig. 10:**
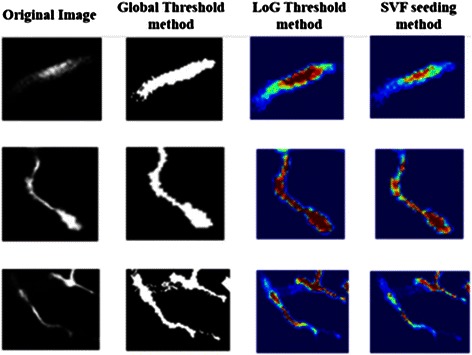
Comparisons among seeding methods for image enhancement. The results of global threshold method lose the contrast information between center and edge; The results of LoG threshold method extend the center region exorbitantly; The results of SVF seeding method can enhance the original volume properly

We also compared the enhancement results from the three methods, and we chose the same cross-section of the neuron volume image to demonstrate that the SVF seeding method can enhance the region around the center line and simultaneously save the contrast information of the tube-like volume. The results are shown in Fig. 10, in which the red part has a higher response value than the blue part. This result suggests that the SVF method can extract the center region better than the other two methods and can enhance the original volume data properly. Furthermore, the better results in both seed detection and SVF volume enhancement can benefit neuron tracing.

### Skeleton tracing

#### A. Tracing accuracy

We adopted the drosophila olfactory axonal datasets (OP_1 to OP_9 from DIADEM challenge) and neocortical layer 1 axons subset 1 datasets (NC_1 to NC_6 from DIADEM challenge) to evaluate the performance of the proposed neuron skeleton tracing method in the term of accuracy. Meanwhile, we compared the SEF-OCS tracing method with some start-of-the-art algorithms, such as the Open Curve Snake tracing method (OCS) [33], Neural Circuit Tracer method (NCT) [34], all-path pruning method (APP) [37], improved all-path pruning method (APP2) [38], distance-field based method (DF) [39], and 3D tubular models based method (TM) [42].

To compare with these methods fairly, we conducted the experiments without any manual interactions and corrections, using the widely used accuracy principle to measure the test results. Similarly, we set the parameters *rad* = 20, *R*_*max=*_16, *R*_*min=*_5, and *t =* 10 in the proposed method, and we chose the better parameters for other six methods according to the features of different datasets. The measured principle is defined as:

12 $$ \begin{array}{l} \Pr ecision= Length(Correct)/ Length(TotalAutomaticTraces)\\ {}\mathrm{R}\mathrm{e} call= Length(Correct)/ Length\left( GoldS \tan dard\right)\end{array} $$

where *Precision* is measured as the proportion of the length of correct traces to the total length of the traces generated by the tested methods, and *Recall* is the proportion of the length of correct traces to the length of the gold standard of adopted datasets.

**Fig. 11:**
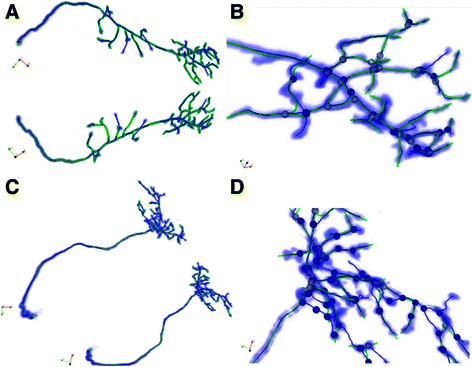
The tracing results of OP_1 and OP_4. **a** The tracing result of OP_1 from multi-view. **b** The magnified result of branch parts of OP_1. **c** The tracing result of OP_4 from multi-view; **d** The magnified result of branch parts of OP_4

Figure 11a and b show the reconstruction results of OP_1, and Fig. 11c and d show the results of OP_4. All of these results were generated by our proposed SEF-Open curve snake method. To illustrate the higher performance of our proposed method, we choose different colors to indicate the differences; the blue lines are the gold standard, and the green lines are the skeleton reconstructed by our method. Additionally, more tracing results of the other datasets are shown in the Additional file 1.

**Fig. 12:**
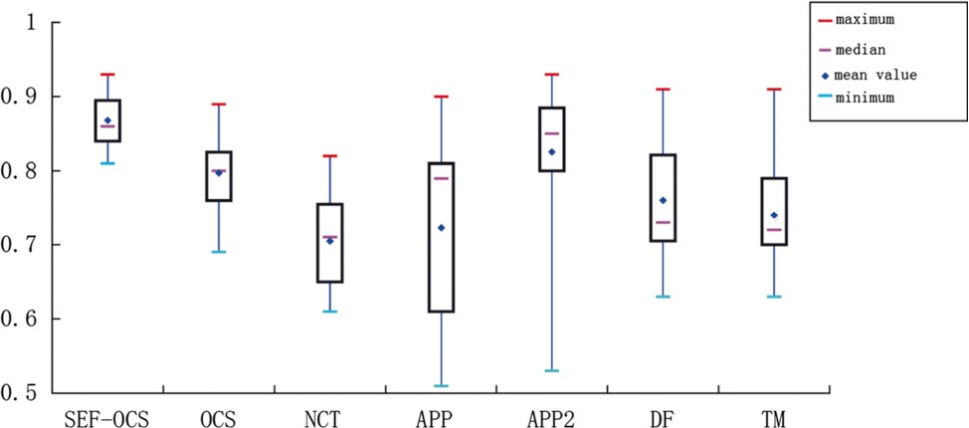
The box plot of DIADEM scores of the different methods for different datasets (OP_1 to OP_9 and NC_1 to NC_6 datasets). The median is the middle pink bar. The box indicates the lower quartile (splits 25 % of lowest data) and the upper quartile (splits 75 % of highest data). The red bar and blue bar are the maximum and minimum values. The blue diamond denotes the mean value of the scores

Table 3 summarizes the comparisons between the OCS and other six methods in terms of precision and recall. We can see that SEF-OCS is far better than other six methods in most datasets in terms of accuracy and recall. In addition, the SEF-OCS outperforms other six methods in average accuracy and recall. We also conducted the DIADEM score test [51] to evaluate the proposed method in the precision of reconstructed neuron topology and compare with the other methods. To the best of our knowledge, due to the various features of different datasets, no methods can get higher DIADEM score in all the datasets automatically. Hence a box plot is adopted to show the DIADEM score distribution of different methods tested in the different datasets. As we can see from Fig. 12, our method can achieve higher DIADEM score in most of the tested datasets and outperforms other six methods in average value (0.87 ± 0.001), median (0.86) and minimum (0.81). This results also proved that the proposed method has higher stability. In order to evaluate the automaticity of the proposed method, we used fixed parameters to test our method in this paper. Actually, The changed parameters can also be tried to get more meaningful tracing results. For instance, when the neuron data includes a big cell body, the bigger *rad* parameter is needed. Additionally, some other methods also can be tried to trace neuron according to the features of different neuron cells. For example, the APP, APP2 and DF methods can achieve better results sometimes.

**Table 3:** Comparisons among different methods with different image datasets in tracing accuracy

Data set	Size	Precision/recall						
		SEF-OCS	OCS	NCT	APP	APP2	DF	TM
OP_1	512*512*60	**0.97**/0.91	0.93/0.85	0.86/0.79	0.73/0.77	0.92/**0.93**	0.82/0.84	0.83/0.87
OP_2	512*512*88	0.93/**0.94**	0.87/0.89	0.81/0.72	**0.97**/0.91	0.95/0.65	0.89/0.74	0.77/0.79
OP_3	512*512*62	0.89/0.94	0.85/0.92	0.79/0.83	**0.94**/0.92	0.85/0.83	0.91/**0.96**	0.91/0.92
OP_4	512*512*67	0.91/0.85	0.92/0.79	0.83/0.76	0.61/0.65	**0.93**/**0.86**	0.72/0.79	0.75/0.79
OP_5	512*512*76	**0.93**/0.94	0.89/**0.95**	0.86/0.81	0.81/0.9	0.85/0.87	0.81/0.84	0.79/0.81
OP_6	512*512*101	0.85/**0.89**	0.79/0.82	0.76/0.73	0.82/0.63	0.89/0.83	**0.93**/0.82	0.67/0.71
OP_7	512*512*71	0.82/**0.91**	0.84/0.86	0.81/0.77	0.75/0.88	**0.91**/0.9	**0.91**/0.86	0.71/0.7
OP_8	512*512*85	0.86/**0.94**	0.73/**0.94**	0.79/0.74	0.7/0.86	**0.93**/0.91	0.83/0.92	0.73/0.74
OP_9	512*512*92	**0.91**/**0.92**	0.87/0.91	0.85/0.81	0.81/0.63	0.83/0.85	0.77/0.91	0.7/0.69
NC_1	512*512*60	0.89/0.87	0.73/0.84	0.79/0.76	**0.96**/**0.93**	0.9/0.89	0.67/0.71	0.71/0.75
NC_2	512*512*33	0.85/0.83	0.81/0.89	0.81/0.79	**0.91**/**0.93**	0.85/0.69	0.87/0.61	0.8/0.81
NC_3	512*512*44	**0.9**/**0.85**	0.72/0.71	0.71/0.69	0.89/**0.85**	0.89/0.7	0.75/0.71	0.73/0.76
NC_4	512*512*51	**0.93**/**0.92**	0.75/0.74	0.67/0.65	0.81/0.85	0.83/0.87	0.82/0.85	0.74/0.75
NC_5	512*512*50	**0.88**/**0.89**	0.7/0.67	0.73/0.65	0.87/0.86	0.84/0.8	0.73/0.7	0.79/0.73
NC_6	512*512*46	0.86/0.85	**0.9**/0.79	0.8/0.64	0.87/**0.89**	0.85/0.81	0.82/0.75	0.81/0.85
Average		**0.89/0.9**	0.82/0.83	0.79/0.74	0.83/0.83	0.88/0.82	0.82/0.8	0.76/0.78

(The best values are highlighted with bold letters)

#### B. Tracing robustness

To verify the robustness of our method, we designed three kinds of experiments. Firstly, the datasets with different levels of signal attenuation were tested. Secondly, the datasets with deferent levels of Gaussian noise were tested. Thirdly, the datasets (checked6_frog_scrippts, checked6_human_culturedcell_Cambridge_in_vitro_confocal_GFP,checked6_human_allen_confocal and checked6_fruitfly_larvae_gmu) from BigNeuron project were also tested using the proposed method.

**Fig. 13:**
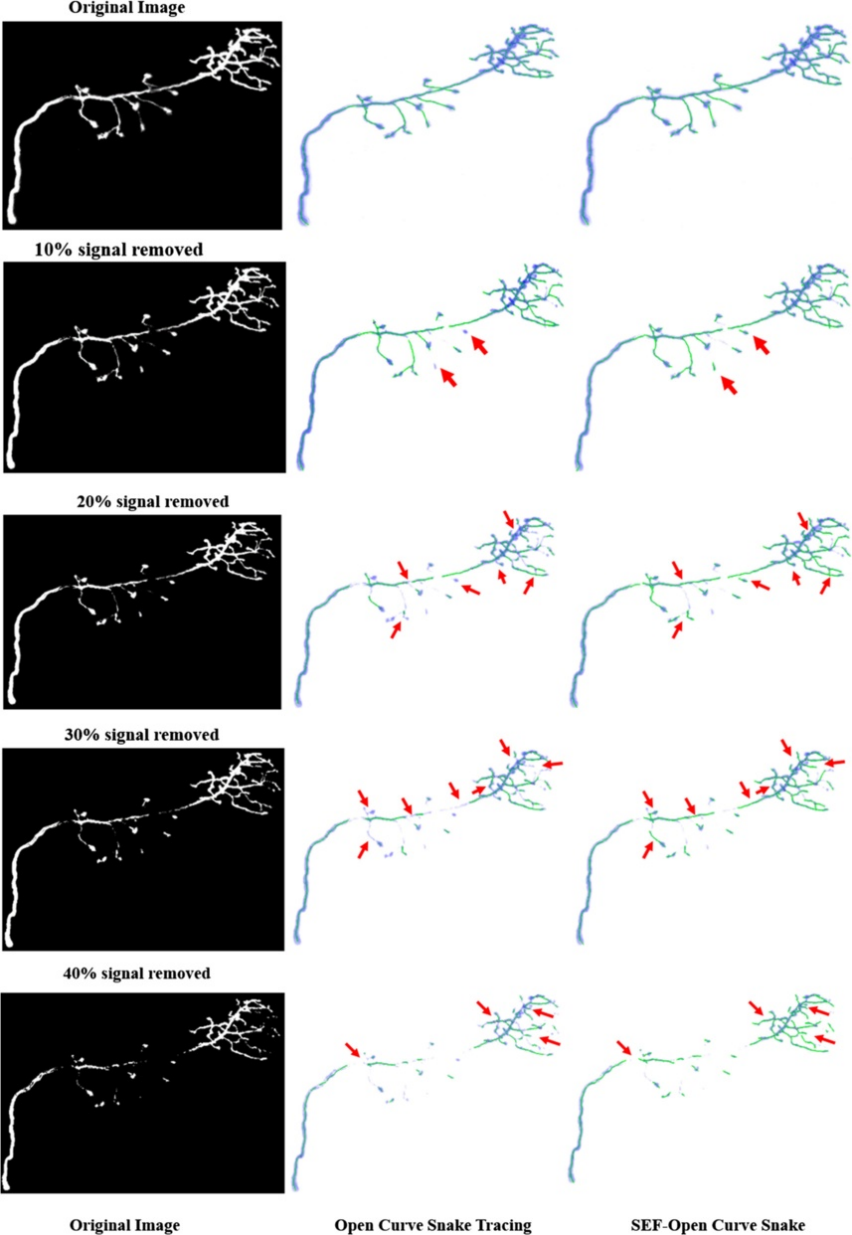
Comparison in signal removed image datasets. To achieve more clear comparison, we follow the same experiment design in [16]

**Fig. 14:**
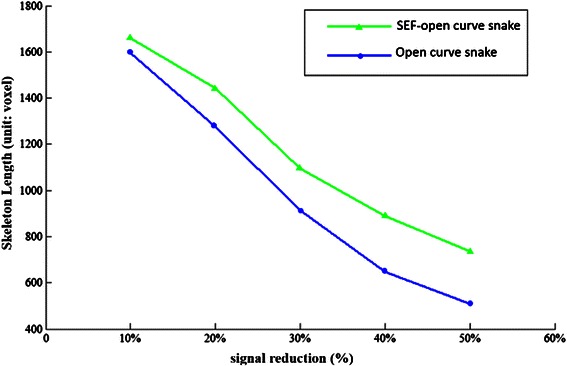
Changes of skeleton length with signal reduction (Unit: Volex)

Firstly, we compared the length of the traced skeleton with OCS in handling image signal reduction. Compared with the traditional robustness test method, which always added Gaussian noise to the original volume, this paper’s test method has a special meaning. Unbalanced light will lead to different levels of signal attenuation in the process of capturing images from a microscope. The OP_1 data set is chosen as an example, and we reduced the image signal from 10 % to 40 %. The content of the neuron images with different degrees of reduction is shown in Fig. 13. In Fig. 14, we list the lengths of the neuron skeleton traced by the two methods for comparison. With the image information reducing from 10 % to 40 %, our method can trace more information than the open curve snake in skeleton length. This result conveys that our method has higher robustness upon image signal reduction. All these results suggest that the proposed method performs better than the OCS method.

**Fig. 15:**
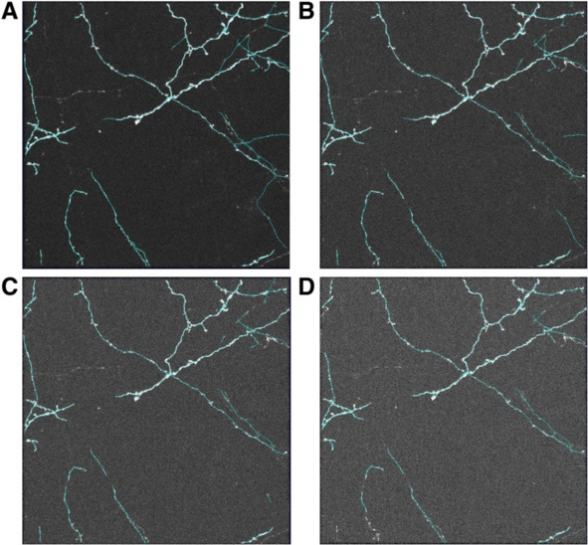
The tracing results of NC_2 dataset with different levels of Gaussian noise. **a** The dataset with a variance of 0.01. **b** The dataset with a variance of 0.02. **c** The dataset with a variance of 0.03. **d** The dataset with a variance of 0.04. To prove the robustness of our method clearly, we follow the similar design of experiment in [39]

Secondly, we tested the robustness of our method using the datasets with different levels of Gaussian noise (The mean is 0 and the variances are 0.01, 0.02, 0.03 and 0.04 respectively.). As we can see from Fig. 15, the blue lines represent the tracing results of the proposed method. The tracing results are tolerable even when the variance is 0.04 and the major branches of neurons are not missed.

**Fig. 16:**
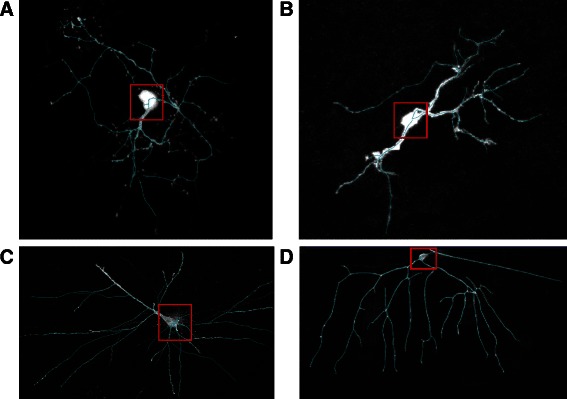
The tracing results of the datasets from the BigNeuron project. **a** The tracing result of the done_err_Recon112012no2-2 data of checked6_frog_scrippts. **b** The tracing result of image 7 data of checked6_human_culturedcell_Cambridge_in_vitro_confocal_GFP. **c** The tracing result of in_house1 data of checked6_human_allen_confocal. **d** The tracing result of done_1_CL-I_X_OREGON_R_ddaD_membrane-GFP data of checked6_fruitfly_larvae_gmu

Thirdly, the datasets from the BigNeuron project were also tested. To my best knowledge, the BigNeuron will be a new trend in this field and most of datasets are challenging. The Fig. 16 shows some tracing results of the datasets of the BigNeuron project. Similarly, the blue lines represent the tracing results of our method. The results are tolerable even these datasets are complex and sometimes include a big cell body (In the Fig. 16, the big cell body is highlighted using the red rectangle). However, *rad* parameter must be set bigger (we set the parameters rad = 35, Rmax = 31, Rmin = 5, and t = 10 in the proposed method) to get better results when the datasets contain a big cell body. Additionally, the APP2 from Vaa3D [43, 44] can also achieve good results automatically when a big cell body exists in the datasets.

### Radius estimation and surface reconstruction

**Fig. 17:**
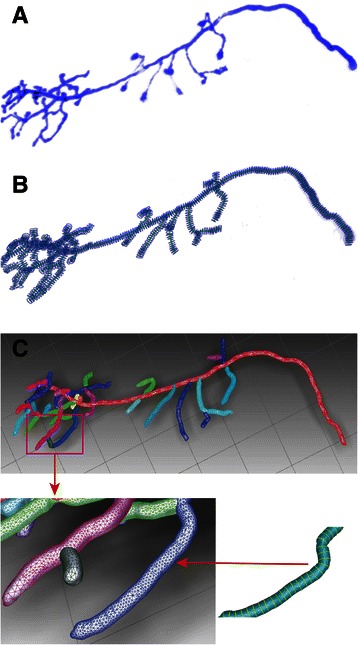
Radius estimation and anatomical reconstruction of OP_1. **a** The original result by volume rendering method. **b** The result of radius estimation by 2D sliding band method, in which the blue contour lines are used to fit the edges of neuron cross-sections. **c** The anatomical reconstruction result based on the contour lines, in which the different branches are labelled with different colors

Adaptive radius estimation and surface reconstruction methods can improve the neuron model, which has branches of varying widths. In radius estimation, the proposed method can fit the edge of the cross-section of the neuron cell. Furthermore, the credibility of radius estimation can be adjusted by the parameter *n*. As we can see from Fig. 5, the higher parameter *n*, the greater the credibility of radius estimation and the higher the computation intensity. Though a higher credibility of radius estimation must be achieved by higher computation intensity, the proposed method has solved the non-circular radius estimation problem mentioned in [33]. Generally, the parameter *n* = 16 is sufficient for most applications. Hence, we set *n* = 16 in the efficiency comparison experiments. Figure 17b shows the radius estimation results of the entire neuron using the proposed method based on the skeleton of the original volume, which is shown in Fig. 17a.

The adopted reconstruction method can interpolate meshes based on contour lines from radius estimation and can also handle branching reconstruction problems efficiently. To illustrate the performance of the proposed framework, the reconstruction result of the most complex data (OP_1) in the OP series data sets is shown in Fig. 17. The morphology of a reconstructed neuron cell of OP_1 was obtained, and the different branches were labelled with different colors during reconstruction.

### Computational efficiency

In the term of automatic computation efficiency, we tested every step of the proposed pipeline and compared with other methods to evaluate the 3D neuron reconstruction efficiency. In addition, we set the parameters rad = 20, Rmax = 16, Rmin = 5, and t = 10 in the proposed method.

As we can see from Table 4, the proposed method is more efficient than the OCS framework, especially in the seeding step because seeding in the OCS framework is based on a complex procedure including graph-cut segmentation and skeleton and seeding point selection. In contrast, our seeding method is more concise and efficient. The higher tracing efficiency also demonstrated the improvements in stretching force in the open snake model and the collision point detection strategy. Additionally, Table 4 also shows that the radius estimation and reconstruction are more efficient in the SEF-OCS framework than in the OCS framework. These results prove that the proposed radius estimation and surface reconstruction methods outperform the corresponding methods in the OCS framework.

**Table 4:** Comparisons with OCS method in the efficiency of the proposed pipeline

Data sets	Size	CPU time (s)								GPU time (s)	
		OCS				SEF-OCS				OCS	SEF-OCS
		S	T	RE	SR	S	T	RE	SR		
OP_1	512*512*60	65	57	42	36	**32**	**33**	**27**	**21**	3.5	**1.5**
OP_2	512*512*88	77	69	53	50	**41**	**47**	**48**	**36**	4.6	**2.5**
OP_3	512*512*62	67	59	49	42	**32**	**34**	**26**	**22**	3.7	**1.7**
OP_4	512*512*67	69	61	46	44	**33**	**34**	**29**	**23**	4.1	**1.9**
OP_5	512*512*76	72	63	59	45	**37**	**39**	**37**	**30**	4.9	**2.3**
OP_6	512*512*101	89	73	67	65	**45**	**57**	**46**	**45**	6.7	**3.5**
OP_7	512*512*71	71	63	53	49	**35**	**36**	**36**	**26**	5.1	**2.1**
OP_8	512*512*85	75	69	58	53	**41**	**43**	**42**	**34**	5.9	**2.4**
OP_9	512*512*92	82	71	59	55	**43**	**52**	**45**	**43**	6.1	**2.9**
Average		74.1	65	54	48.8	**37.7**	**41.7**	**37.3**	**31.1**	4.96	**2.31**

(S: Seeding; T: Tracing; RE: Radius Estimation; SR: Surface Reconstruction; The best values are highlighted with bold letters)

To test the ability of parallel computation, we also developed our CUDA implementation for the four main steps. The computation time is shown in the Table 4. We can see that the proposed method is faster than OCS method in the same parallel environment. In addition, the average speedup ratio of SEF-OCS can achieve 63.94, which is higher than OCS’s average speedup ratio 48.8. This also demonstrates that the proposed method has higher parallel computation ability.

Table 5 shows the efficiency comparison results with some other methods from Table 3. Actually, most of neuron reconstruction methods don’t contain the surface reconstruction procedure. Hence, we conducted the comparison experiments in another way. We cut down the computational cost of the surface reconstruction step in order to carry out the comparison among different methods fairly. What’s more, the experiments are conducted three times for every method corresponding to every data set to avoid the errors from the operation system environment. The average results of three times are shown in Table 5. The comparison results show that the proposed method achieve the lowest average computational cost. We also can see that the computational cost of our method mainly depend on the size of the data sets and will realize higher efficiency with the development of computation parallel capacity.

**Table 5:** Comparisons in the efficiency of neuron tracing

Data sets	Size	SEF-OCS (s)	NCT (s)	APP (s)	APP2 (s)	DF (s)	TM (s)
OP_1	512*512*60	1.1	191.5	5.3	**0.6**	9.2	163.5
OP_2	512*512*88	1.5	237.1	9.0	**0.9**	17.1	251
OP_3	512*512*62	1.2	178.3	14.0	**0.6**	10.5	165.7
OP_4	512*512*67	1.4	165.2	5.6	**0.7**	13.7	177.6
OP_5	512*512*76	1.7	233.5	28.5	**0.6**	15	201.2
OP_6	512*512*101	1.9	277.6	7.6	**0.7**	20	255.6
OP_7	512*512*71	1.4	136.7	16.4	**0.6**	14.3	182.1
OP_8	512*512*85	1.5	175.9	36.4	**0.7**	16.7	231.1
OP_9	512*512*92	2	256.3	7.8	**0.7**	17.9	245.5
NC_1	512*512*60	**1.2**	125.6	27.0	2.3	19.8	152.3
NC_2	512*512*33	**0.5**	57.6	11.7	1.0	6.7	81.2
NC_3	512*512*44	**0.7**	76.5	14.9	1.2	12.3	112.2
NC_4	512*512*51	**1.1**	91.3	21.7	2.5	13.5	131.7
NC_5	512*512*50	**0.9**	76.9	43.1	4.6	9.1	125.6
NC_6	512*512*46	**0.9**	57.2	21.1	2.1	7.5	121.9
Average		**1.27**	155.8	18.01	1.32	13.55	173.21

(The best values are highlighted with bold letters)

## Conclusions

Neuron cell anatomy structure reconstruction plays a very important role in the field of neurology. In this paper, we have developed a new neuron tracing framework, which is based on a sliding filter. We improved every step of the traditional framework compared to the OCS framework. First, given a non-circular cross-section of a neuron, the sliding filter method was introduced to the proposed seeding method (SVF) and radius estimation method (SBF), which is critical for accurately tracing skeletons and reconstructing real morphology. Second, on the basis of better seeding results, the traditional open curve snake model was improved by introducing a new external force to aid the curve evolution for neuron skeleton tracing and a new strategy for collision detection. Finally, a surface reconstruction method based on contour lines was used to generate whole neuron morphology.

A series of experiments have proved that the proposed framework has higher efficiency, stability and robustness in tracing accuracy. In addition, the proposed estimation method and adopted neuron reconstruction method can obtain more accurate neuron morphology, which is meaningful for future works such as simulation and analysis of neuron function in the field of neuroscience research.

### Availability of supporting data

The source code can be available in the website [52]. The OP and NC datasets come from DIADEM challenge project, it can be downloaded from [53]. The checked6_frog_scrippts datasets, the checked6_human_culturedcell_Cambridge_in_vitro_confocal_GFP datasets, the checked6_human_allen_confocal datasets and the checked6_fruitfly_larvae_gmu datasets are available in the BigNeuron project whose website is [54].

## Additional file

Additional file 1: **This document includes additional figures not included in the paper.** Some other tracing results are shown in the supplementary material (12 figures). S1-S7 are some tracing results of BigNeuron datasets. S8-S9 are some other tracing results of NC datasets. S10-S12 are some other tracing results of OP datasets. (PDF 1439 kb)

## Abbreviations

VCI: Voxel Convergence Index
SBF: Sliding Band Filter
SVF: Sliding Volume Filter
SEF-OCS: SVF external force open curve snake
OCS: Open Curve Snake
MA: Medial axes
NCT: Neural Circuit Tracer method
APP: All-path pruning method
APP2: Improved all-path pruning method
DF: Distance-field based method
TM: 3D tubular models based method
S: Seeding
T: Tracing
RE: Radius estimation
SR: Surface reconstruction
