# Supplementary material for: Neuron anatomy structure reconstruction based on a sliding filter
Source: BMC Bioinformatics. 2015 Oct 24;16:342. doi: 10.1186/s12859-015-0780-0 (PMC4619512; doi:10.1186/s12859-015-0780-0)
Supplement: Supplementary file 1 — This document includes additional figures not included in the paper. Some other tracing results are shown in the supplementary material (12 figures). S1-S7 are some tracing results of BigNeuron datasets. S8-S9 are some other tracing results of NC datasets. S10-S12 are some other tracing results of OP datasets. (PDF 1439 kb) [file 12859_2015_780_MOESM1_ESM.pdf]

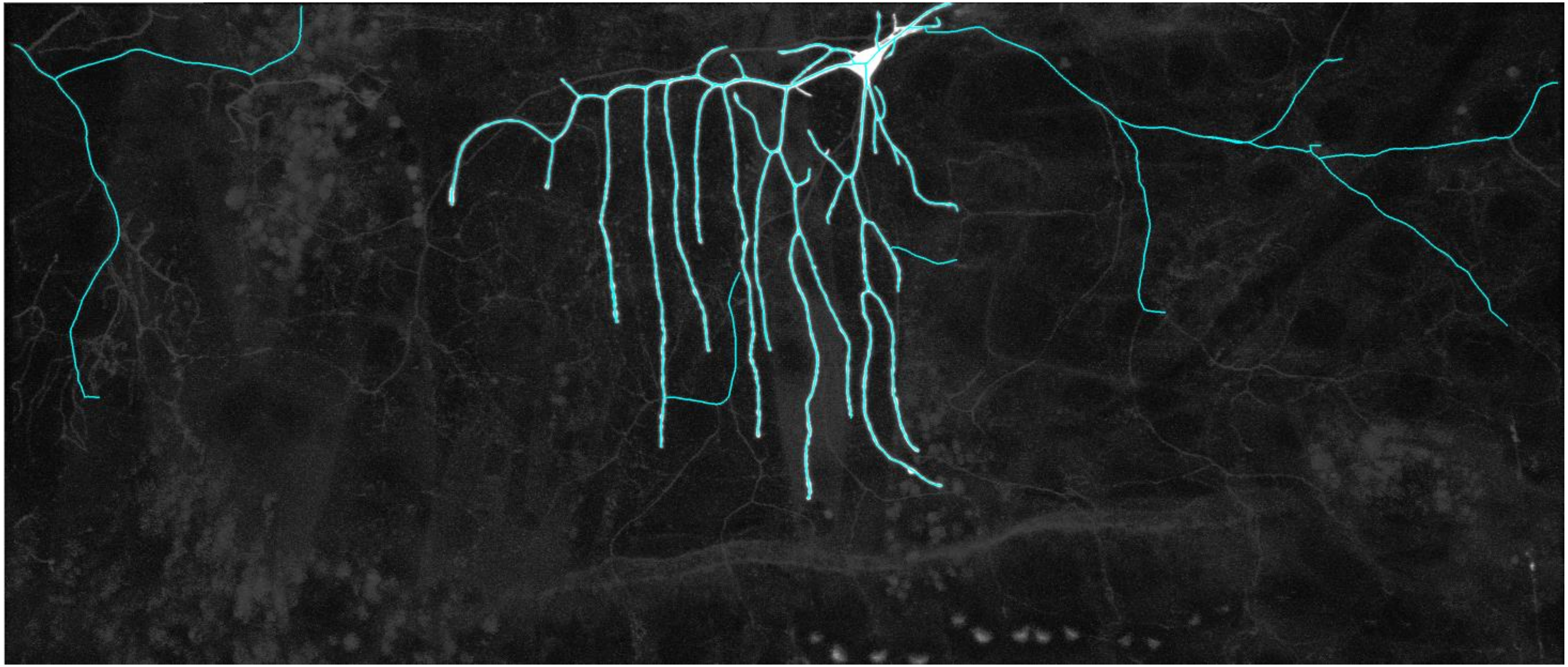

S1. The tracing result of the done_1_CL-I_X_OREGON_R_ddaE_membrane-GFP dataset of checked6_fruitfly_larvae_gmu from the BigNeuron project.

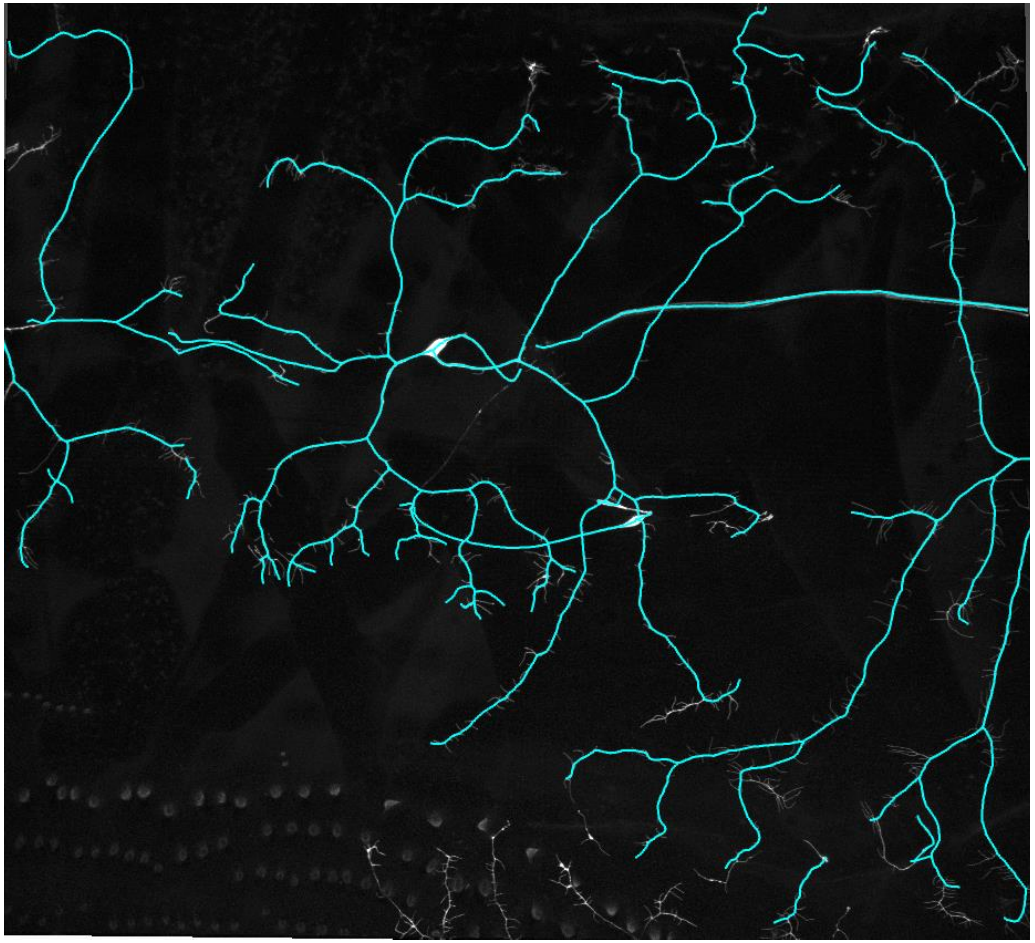

S2. The tracing result of the done_1_CL-III_X_LifeActRuby_vpda_membrane-GFP_actin-LifeActRuby.czi-C_1 dataset of checked6_fruitfly_larvae_gmu from the BigNeuron project.

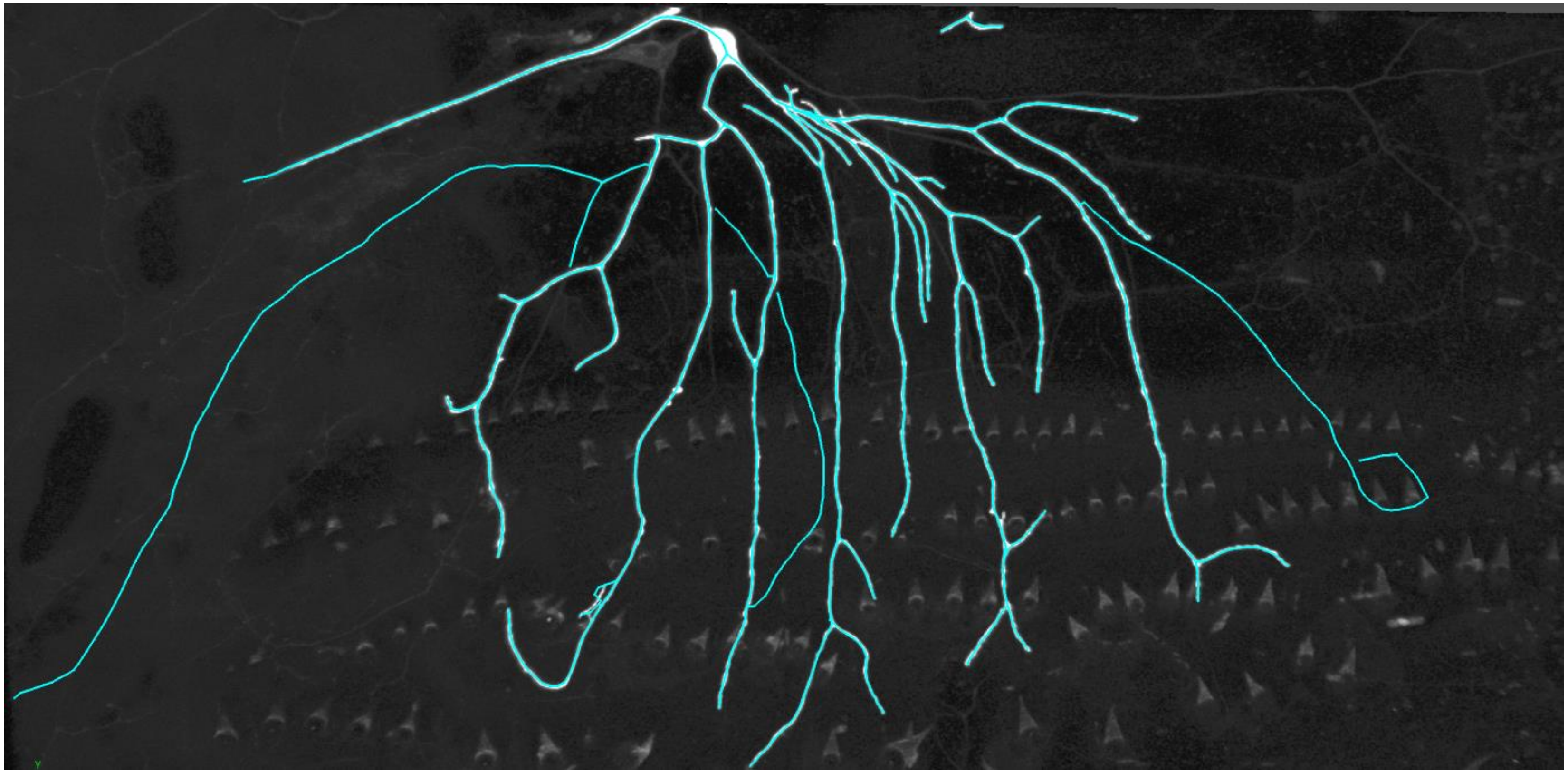

S3. The tracing result of the done_2_CL-I_Membrane-GFP_X_F-Actin-Red_ddaD_Membrane-GFP_F-Actin-Red.czi_C_1 dataset of checked6_fruitfly_larvae_gmu from the BigNeuron project.

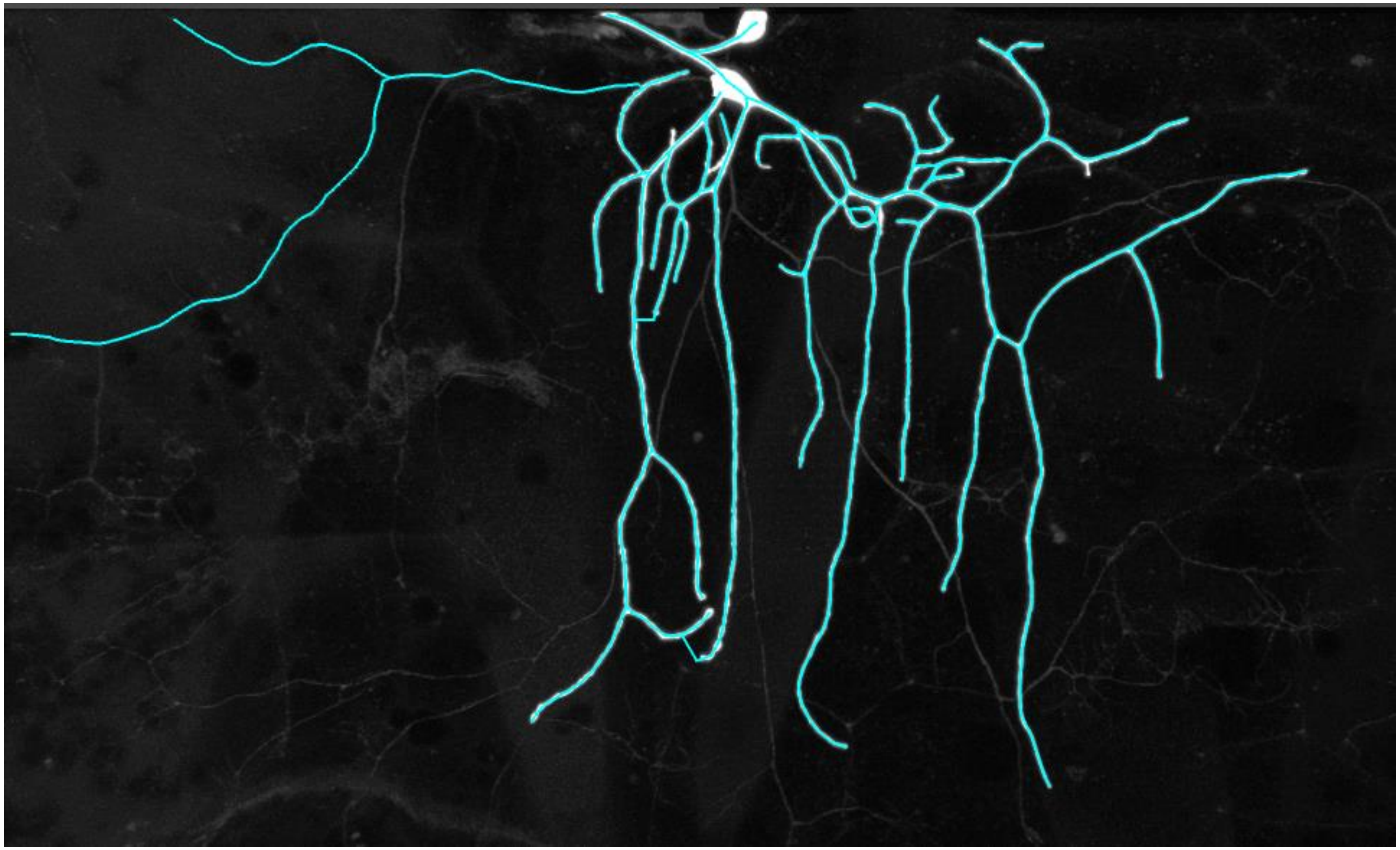

S4. The tracing result of the done_2_CL-I_Membrane-GFP_X_F-Actin-Red_ddaE_Membrane-GFP_F-Actin-Red.czi_C_1 dataset of checked6_fruitfly_larvae_gmu from the BigNeuron project.

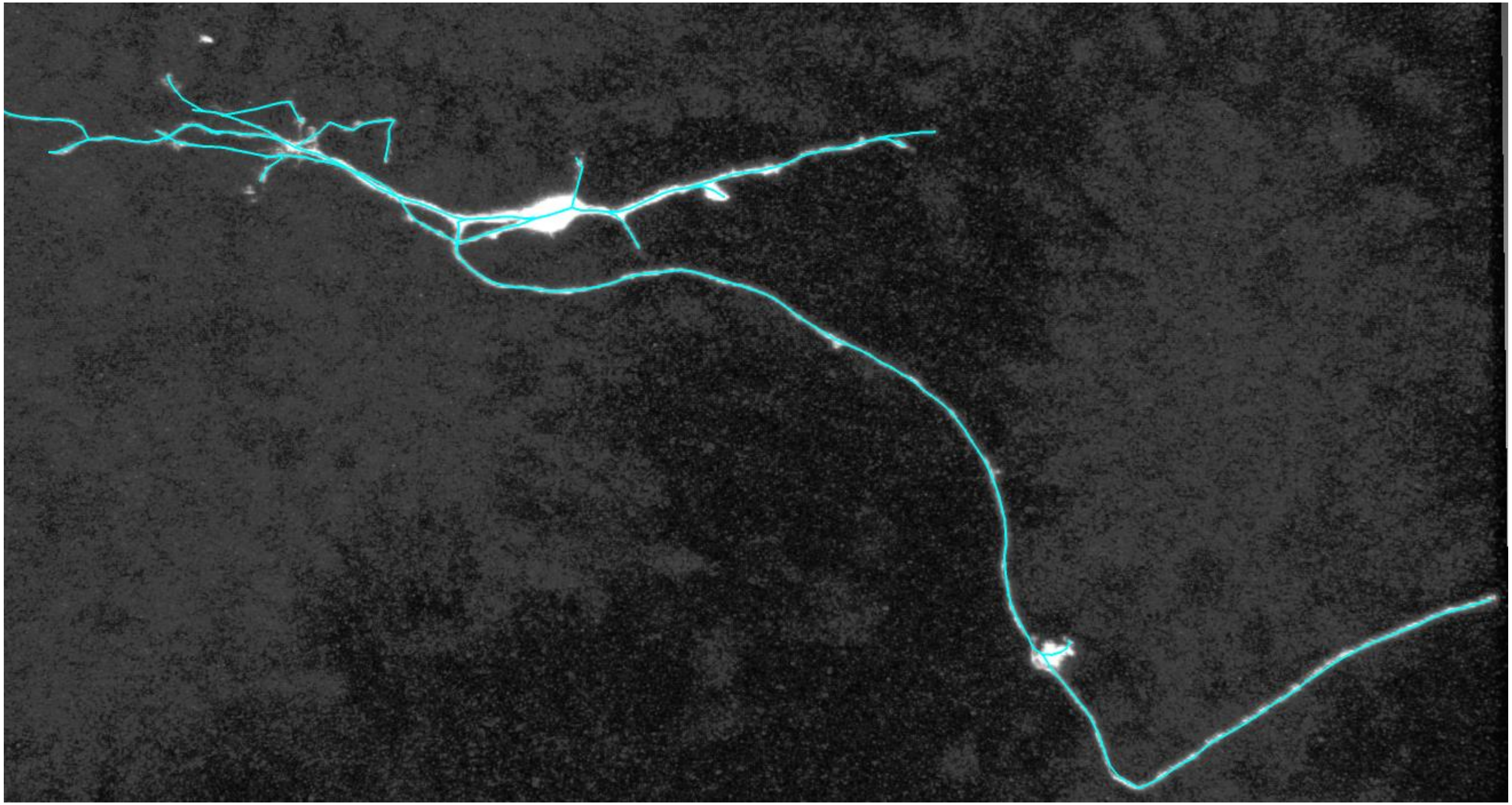

S5. The tracing result of the image 4 dataset of checked6_human_culturedcell_Cambridge_in_vitro_confocal_GFP from the BigNeuron project.

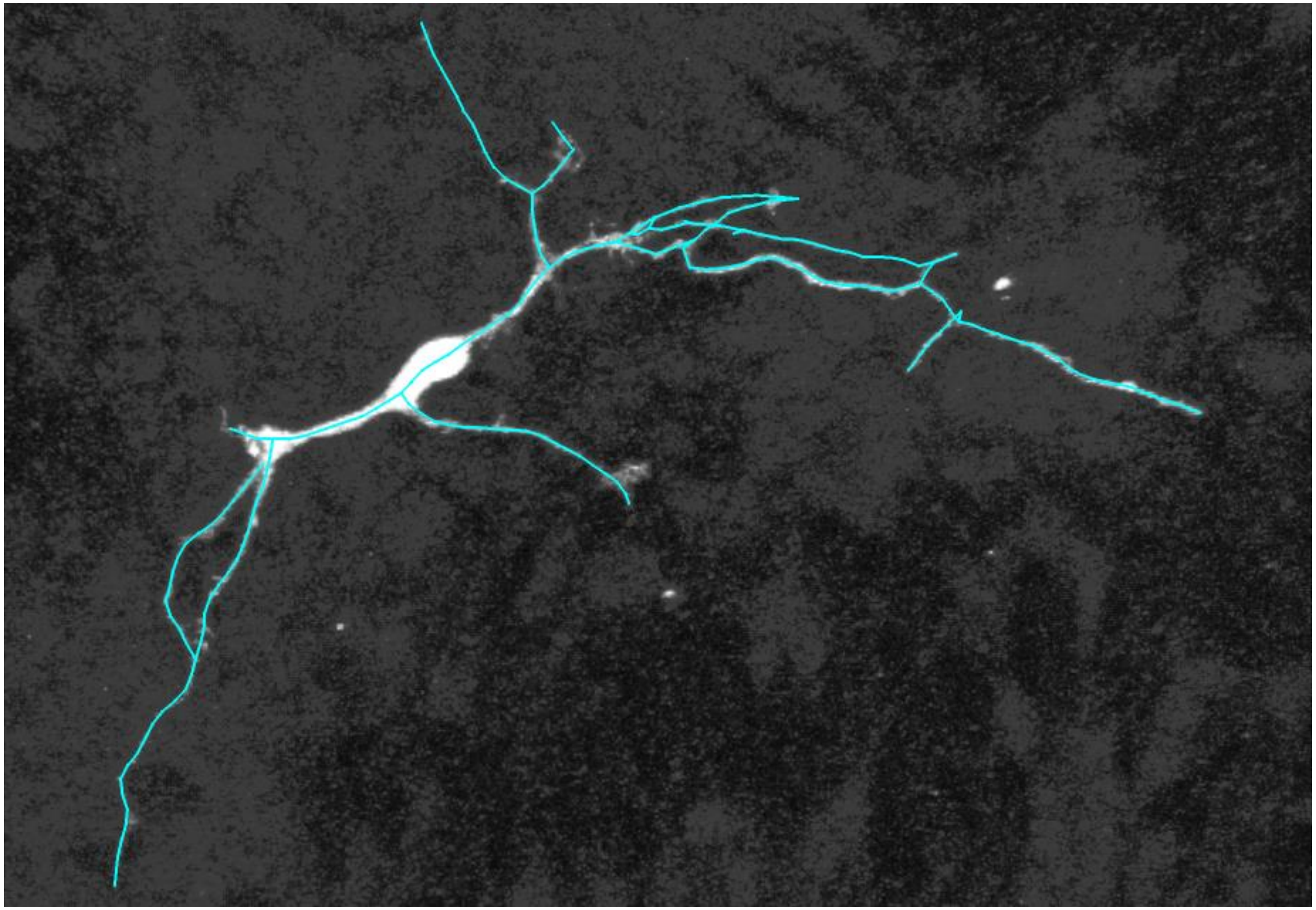

S6. The tracing result of the image 5 dataset of checked6_human_culturedcell_Cambridge_in_vitro_confocal_GFP from the BigNeuron project.

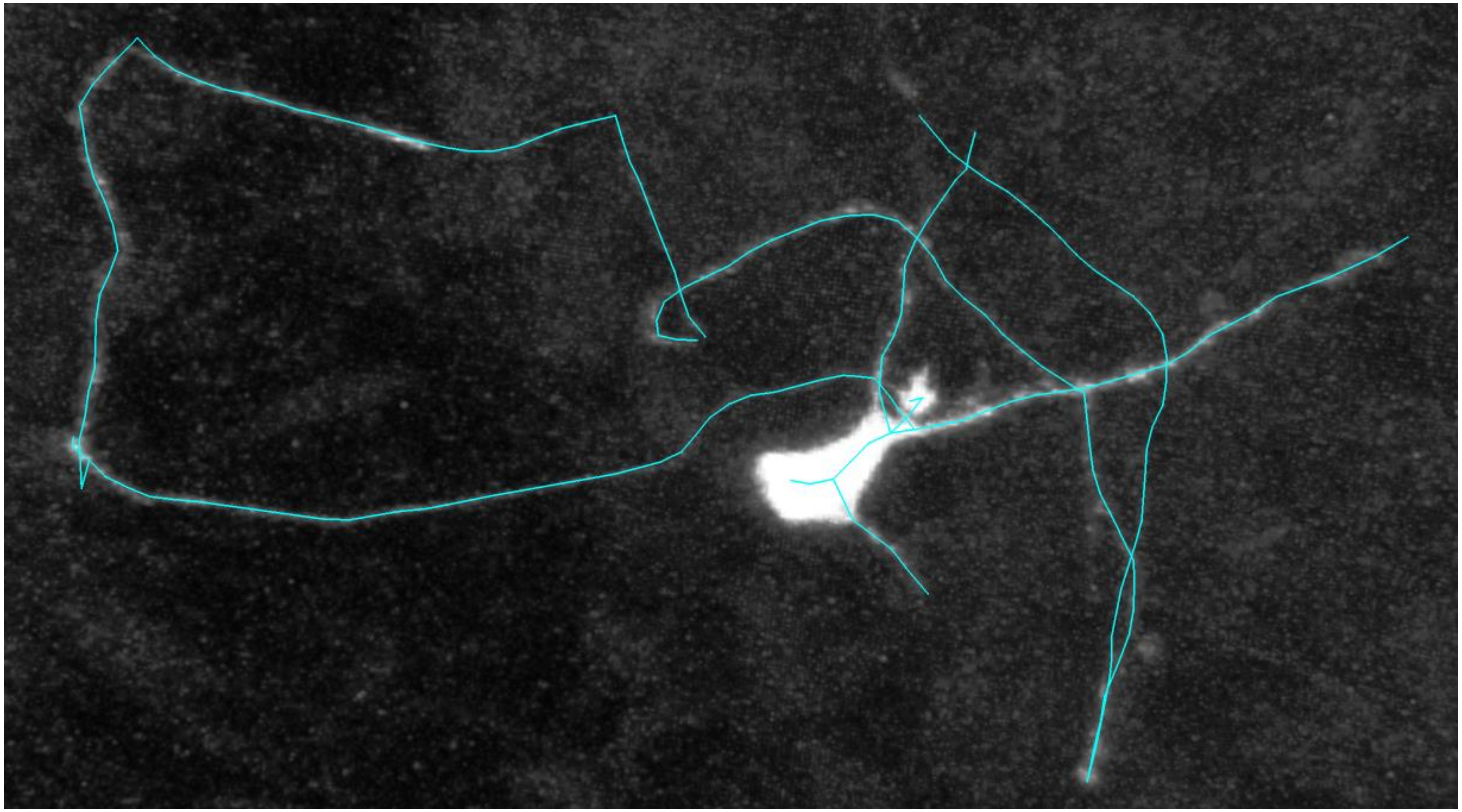

S7. The tracing result of the image 10 dataset of checked6_human_culturedcell_Cambridge_in_vitro_confocal_GFP from the BigNeuron project.

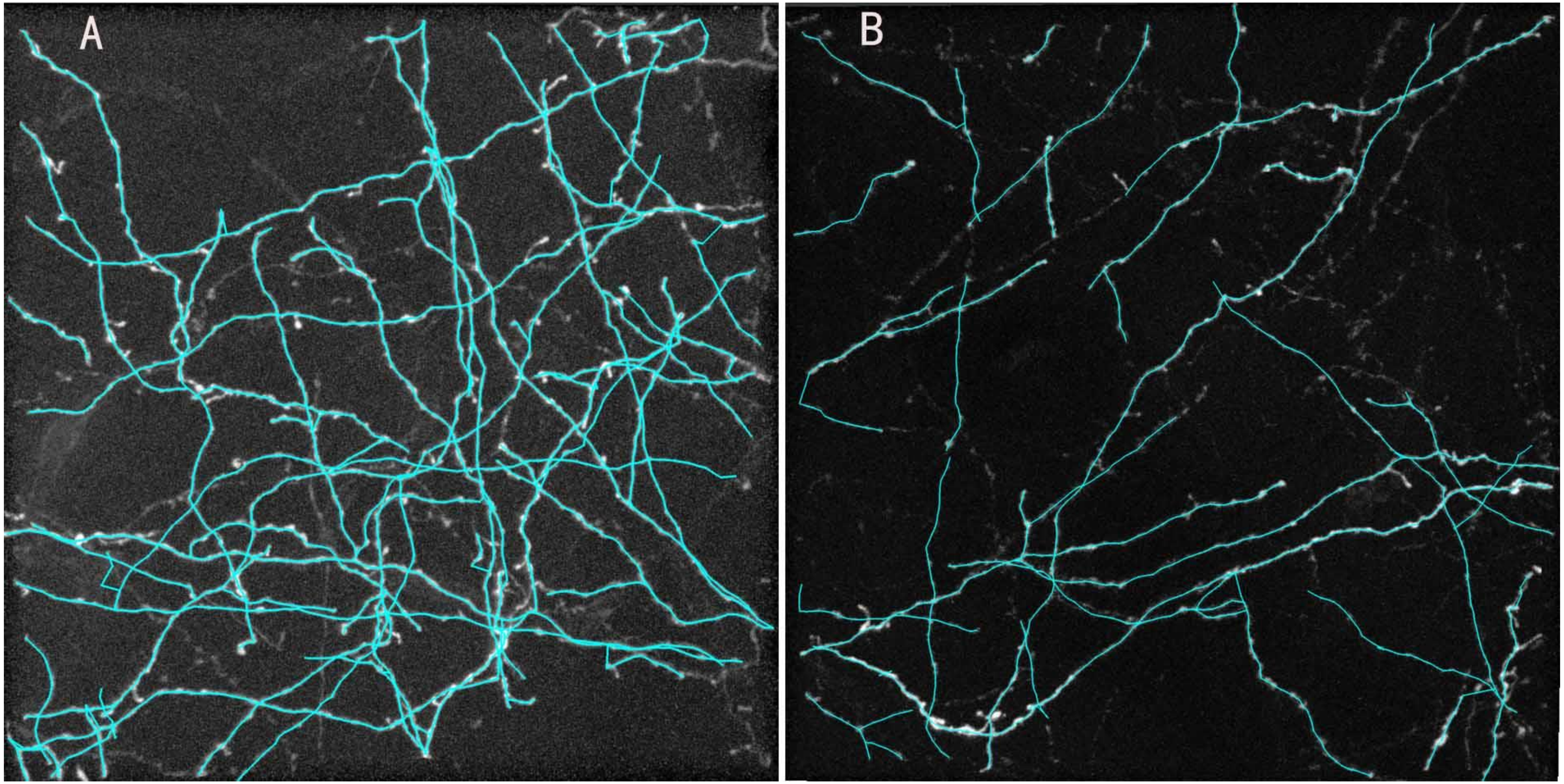

S8. A) The tracing result of the NC1 datasets from the DIADEM challenge. B) The tracing result of the NC3 datasets from the DIADEM challenge.

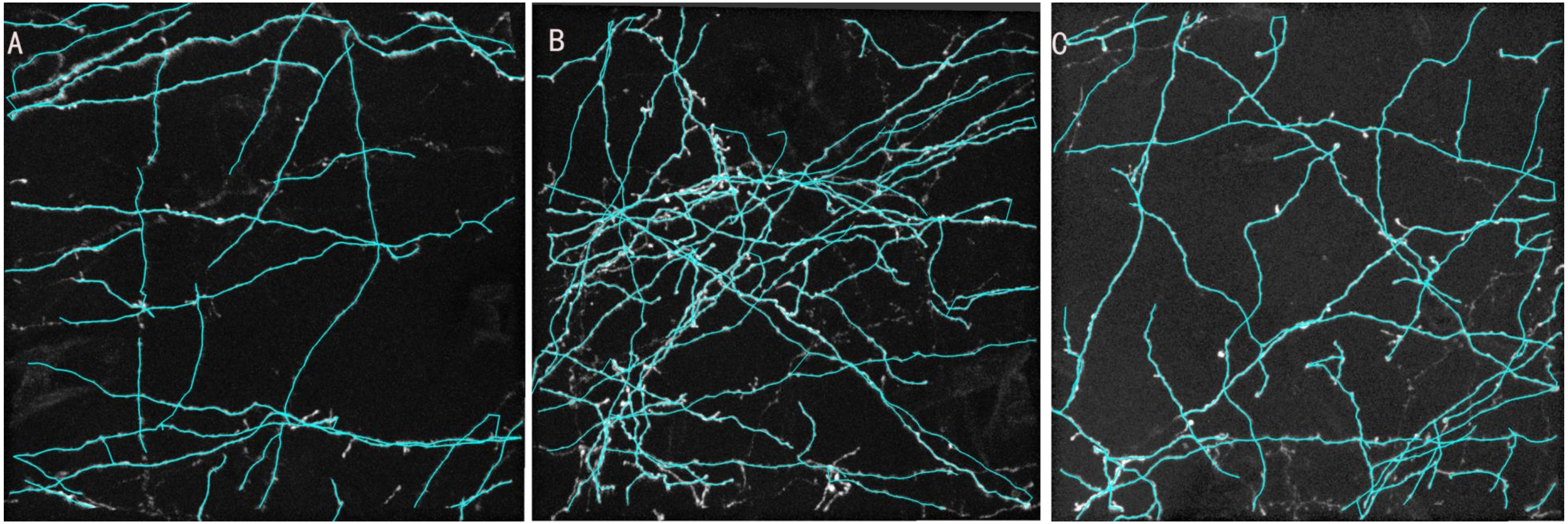

S9. A) The tracing result of the NC4 datasets from the DIADEM challenge. B) The tracing result of the NC5 datasets from the DIADEM challenge. C) The tracing result of the NC6 datasets from the DIADEM challenge.

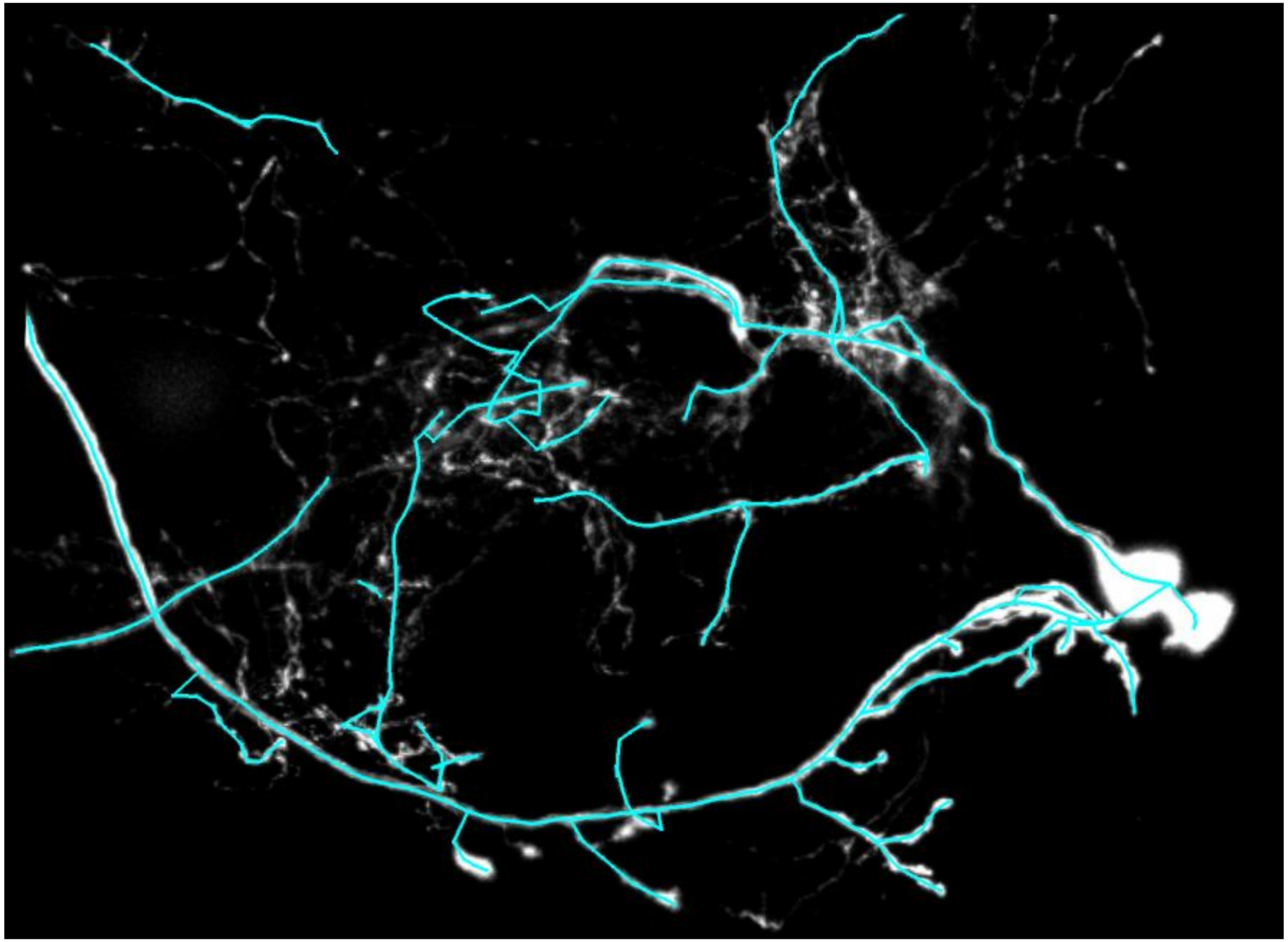

S10. The tracing result of the OP2 datasets from the DIADEM challenge.

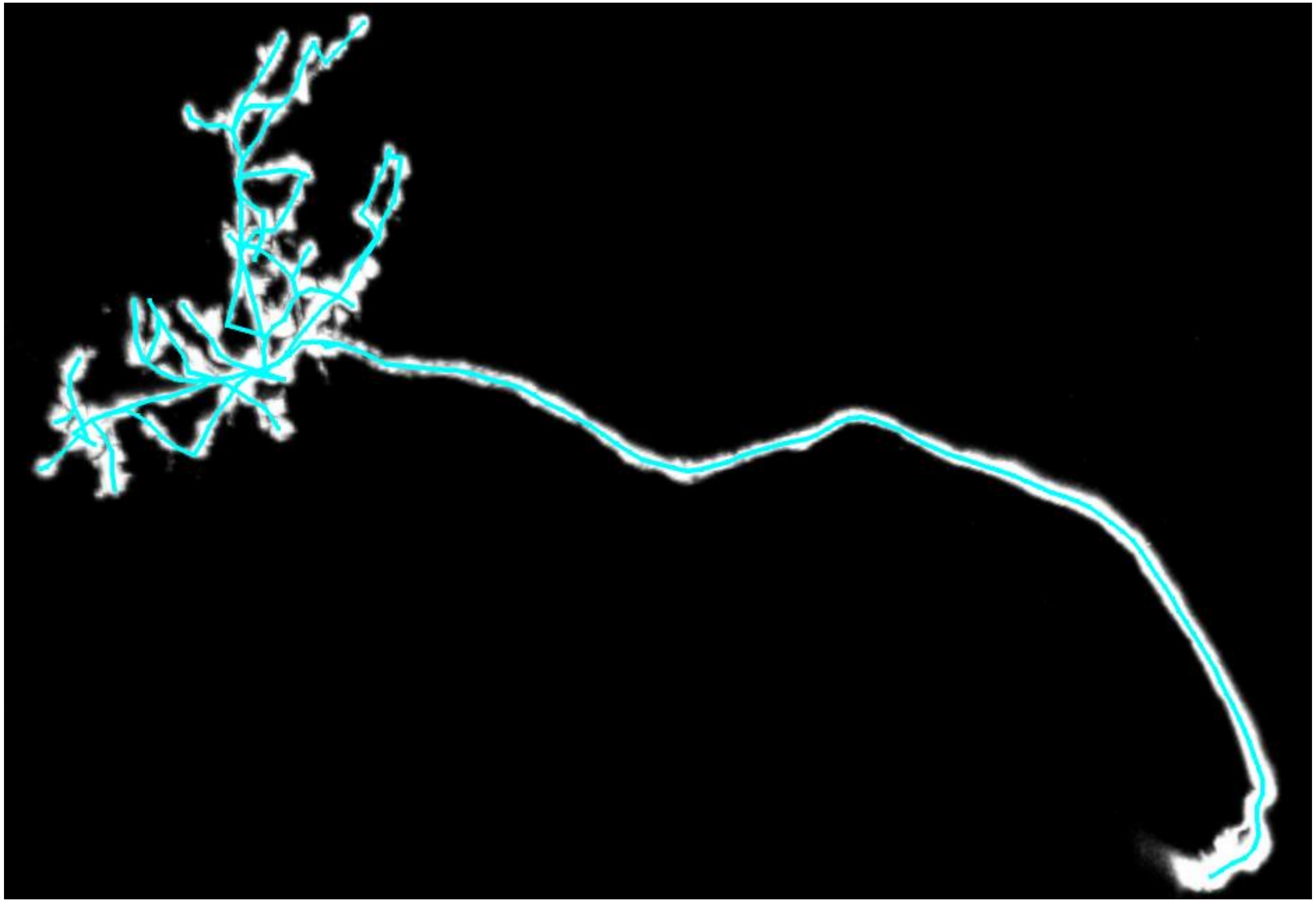

S11. The tracing result of the OP4 datasets from the DIADEM challenge.

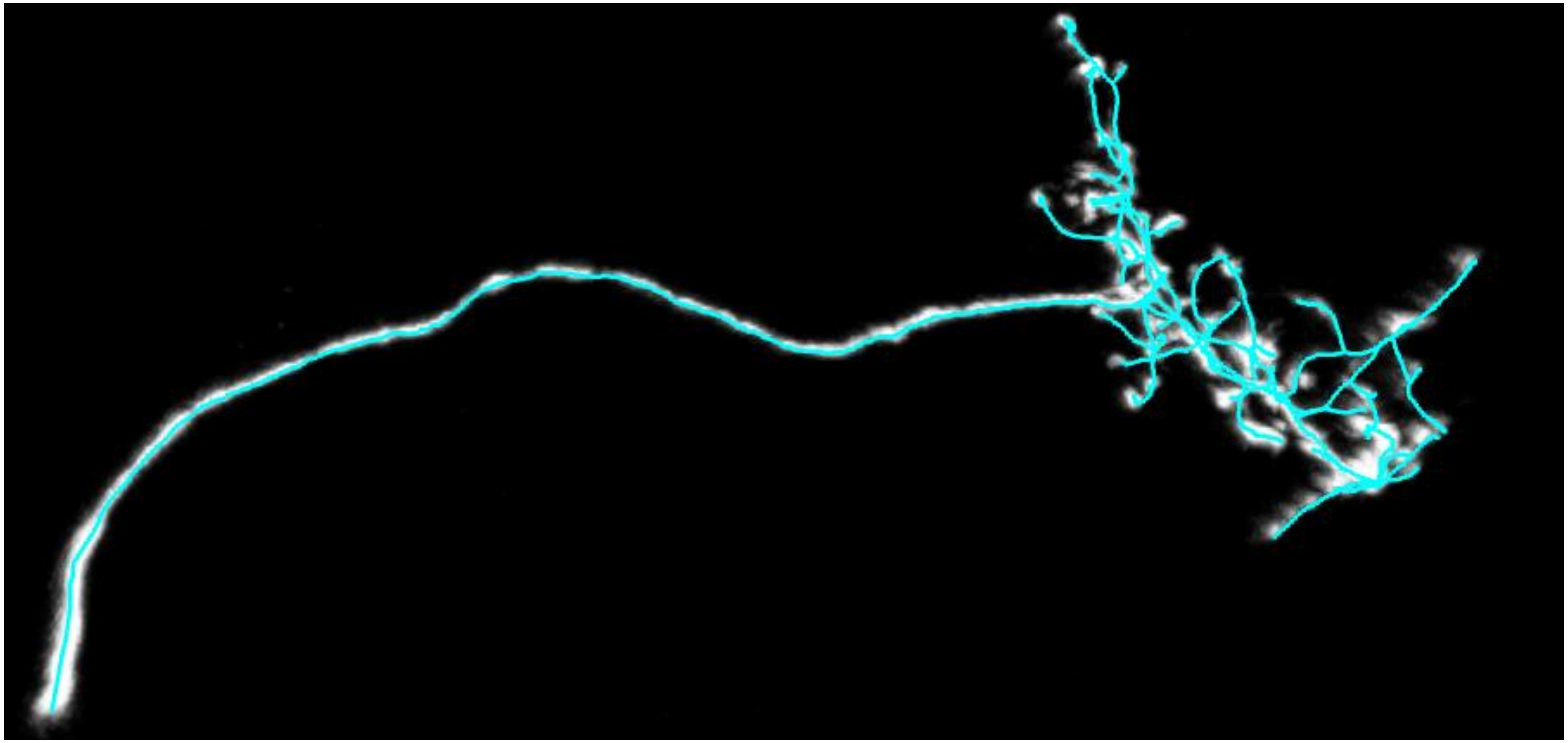

S12. The tracing result of the OP9 datasets from the DIADEM challenge.
